# Routine Laboratory Biomarkers for Survival Prediction After Limb-Salvage Surgery in Osteosarcoma: Development of a LASSO-Cox Prognostic Model and Internal Validation

**DOI:** 10.3390/jcm15186962

**Published:** 2026-09-08

**Authors:** Guangqiang Zhang, Juntan Li, Ting Chen, Jiatong Li, Kai Sun, Lili Guan, Guanning Shang

**Affiliations:** Department of Orthopedics, Shengjing Affiliated Hospital of China Medical University, No. 36 Sanhao Street, Heping District, Shenyang 110036, China; gqzhang2025@cmu.edu.cn (G.Z.); lijt@cmu.edu.cn (J.L.); chenting88726@163.com (T.C.); jtli1995@163.com (J.L.); sunk@sj-hospital.org (K.S.)

**Keywords:** osteosarcoma, limb-salvage surgery, prognostic model, Cox regression

## Abstract

**Background**: Despite advances in treatment strategies, osteosarcoma patients undergoing limb-salvage surgery (LSS) still face a considerable risk of recurrence and unsatisfactory long-term survival. Therefore, reliable pretreatment baseline prognostic assessment tools are needed to improve postoperative risk stratification and guide individualized treatment. **Methods**: This single-center retrospective prediction model development study analyzed 118 patients with pathologically confirmed osteosarcoma treated at Shengjing Hospital, China Medical University, between January 2019 and January 2025. After applying predefined inclusion and exclusion criteria, 81 patients were included in the final analysis. All predictors were defined as before-treatment baseline measurements at the diagnosis stage: alkaline phosphatase (ALP) and lactate dehydrogenase (LDH) were serum biochemical markers, whereas fibrinogen (FIB) and D-dimer were plasma coagulation-related markers. Ki-67 index, age, sex, tumor size, and lung metastasis status were also assessed at this before-treatment baseline. A diagnosis-stage baseline prognostic model for overall survival (OS) among patients undergoing LSS was subsequently constructed. Internal validation of model performance was performed using bootstrap resampling only, with no external validation performed. **Results**: Kaplan–Meier analysis demonstrated 1-, 3-, and 5-year survival rates of 87.3%, 74.3%, and 64.1%, respectively. Survival curve analysis revealed that patients with elevated levels of all four laboratory biomarkers were associated with shorter OS. Multivariate Cox regression analysis revealed that baseline ALP remained independently associated with OS, while FIB showed borderline significance (*p* = 0.050). The final OS prognostic model incorporated ALP, LDH, FIB, and D-dimer. The apparent Harrell’s concordance index (C-index) of the model was 0.838 for the full dataset, with an optimism-corrected C-index of 0.780 after Bootstrap internal validation. The area under the receiver operating characteristic curve (AUC) of the model for predicting 1-, 3-, and 5-year OS in the full dataset was 0.831, 0.864, and 0.871, respectively; the optimism-corrected AUC values after Bootstrap validation were 0.771, 0.810, and 0.814. **Conclusions**: In this single-center retrospective cohort, higher baseline levels of ALP, LDH, FIB, and D-dimer were associated with adverse clinicopathological characteristics and poorer survival outcomes in patients with osteosarcoma. The LASSO-Cox-based prognostic model showed promising discrimination and calibration after internal Bootstrap validation. However, given the limited sample size and absence of external validation, these findings should be considered preliminary, and prospective multicenter external validation is required before the model can be considered for routine clinical implementation.

## 1. Introduction

Osteosarcoma is the most common highly malignant primary bone tumor in adolescents and young adults, accounting for approximately 20–30% of all primary bone tumors [1,2]. Over the past four decades, treatment strategies for osteosarcoma have evolved substantially. Historically, amputation and rotationplasty were the primary surgical approaches. However, with the development of effective neoadjuvant chemotherapy regimens and advances in LSS techniques, this approach has gradually become the preferred treatment modality for most patients with osteosarcoma [3]. However, amputation remains unavoidable in specific clinical scenarios, particularly in patients with extensive tumor invasion of major neurovascular structures, poor prognosis, or disease progression despite neoadjuvant chemotherapy. Although rotationplasty, a specialized form of amputation, can preserve partial weight-bearing function, it often results in an abnormal postoperative limb appearance and requires prolonged rehabilitation, leading to limited patient acceptance [4]. LSS aims to maximize preservation of limb function and appearance while ensuring complete tumor resection with negative margins (R0 resection). Limb integrity is subsequently restored using prosthetic or biological reconstruction techniques. Compared with amputation, it has been shown to significantly improve postoperative functional outcomes and quality of life while maintaining comparable oncological safety [5]. Currently, multimodal comprehensive treatment—consisting of neoadjuvant chemotherapy, surgery, and postoperative adjuvant chemotherapy—has become the standard of care for osteosarcoma. Although this strategy has improved the 5-year survival rate by approximately 20% compared with amputation alone, OS remains between 60% and 70% [6,7]. Distant metastasis, particularly pulmonary metastasis, and local recurrence remain the primary causes of treatment failure [8]. However, there is currently a lack of simple and convenient prognostic tools specifically designed for this patient population. Therefore, identifying clinically accessible prognostic biomarkers is essential for improving risk stratification, refining prognostic evaluation systems, and facilitating personalized treatment strategies [9].

Preoperative laboratory biomarkers provide a convenient and quantitative method for prognostic assessment in osteosarcoma patients undergoing LSS [10]. Specifically, ALP is closely associated with abnormal osteogenic activity in osteosarcoma cells, and elevated ALP levels have been linked to increased tumor burden and extensive bone involvement [11,12]. LDH, a key enzyme in the glycolytic pathway, reflects tumor metabolic activity; elevated LDH levels often indicate rapid tumor proliferation and aggressive biological behavior [13]. However, its independent prognostic value in this population remains uncertain. D-dimer and FIB, important components of the coagulation-fibrinolysis system, have also been reported as potential biomarkers in osteosarcoma. Huang et al. demonstrated that these markers may predict chemotherapy response in osteosarcoma patients [14,15], and additional studies have shown prognostic significance in metastatic osteosarcoma patients receiving second-line chemotherapy [16]. Moreover, elevated FIB levels have been associated with tumor invasion and metastasis in several malignancies, including lung cancer [17,18]. However, the combined prognostic value of ALP, LDH, FIB, and D-dimer in this surgical population has not been systematically investigated.

Previous prognostic studies in osteosarcoma have investigated a wide range of clinicopathological, molecular, and imaging-based factors. More recent prediction models have further incorporated biomarkers such as Ki-67 and EZRIN expression and 18F-FDG PET/CT-derived metabolic parameters [19]. Although such multidimensional models may provide more comprehensive prognostic information, their reliance on specialized imaging or pathological assessments may limit routine applicability. By validating the combined predictive value of four readily available laboratory biomarkers, our study aims to develop a convenient and cost-effective prognostic assessment tool to support future risk-informed clinical assessment and individualized follow-up discussions for osteosarcoma patients undergoing LSS.

**Figure 1:**
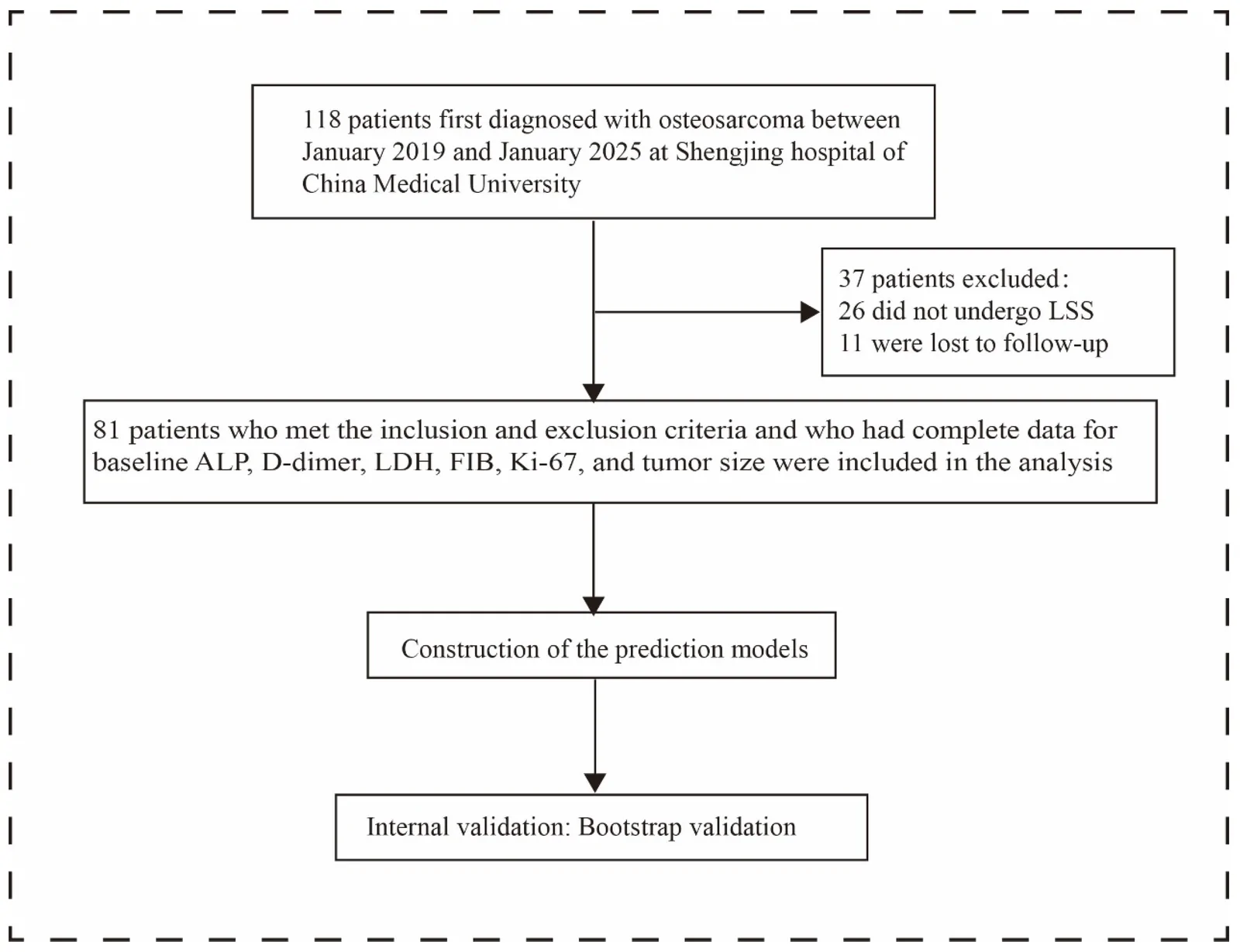
Patient selection and model development flowchart. All baseline predictors were measured at the time of diagnosis, before any treatment. After neoadjuvant chemotherapy, eligible patients underwent limb-salvage surgery. Follow-up started on the date of pathological biopsy confirmation and continued until death or last known alive (right-censored). The prognostic model was developed and internally validated using Bootstrap resampling.

The primary objective of this study was to develop and internally validate a prognostic model for overall survival based on routinely available baseline laboratory biomarkers in patients with osteosarcoma undergoing limb-salvage surgery. As a secondary exploratory objective, we also evaluated postoperative functional recovery and health-related quality of life. The overall study design and patient selection process are summarized in Figure 1.

## 2. Materials and Methods

### 2.1. Patients

This retrospective study reviewed 118 patients with pathologically confirmed osteosarcoma treated at Shengjing Hospital of China Medical University between January 2019 and January 2025. Of these, 92 patients underwent limb-salvage surgery. Follow-up was measured from the date of pathological diagnosis by needle biopsy. Median follow-up was estimated using the reverse Kaplan–Meier method. Inclusion criteria were (1) a definitive diagnosis of osteosarcoma based on pathological examination by two experienced pathologists; (2) complete and traceable clinical records; and (3) a follow-up duration of ≥3 months with complete follow-up data. Exclusion criteria were (1) refusal or inability to cooperate with follow-up; (2) incomplete follow-up data or missing key information; and (3) patients who underwent primary amputation as the initial surgical procedure, rather than LSS. After application of the eligibility criteria, 81 patients were included in the final analysis. All patients underwent pathological examination for definitive diagnosis and tumor staging and had complete clinical records. Demographic characteristics (age, sex), clinical and pathological tumor characteristics, and follow-up data were collected, and baseline levels of four laboratory biomarkers were measured (Table 1). All patients had newly diagnosed primary osteosarcoma without recurrent disease at enrollment. All received the standardized neoadjuvant chemotherapy regimen, while subsequent treatment was modified according to chemotherapy response and metastatic status.

This study was approved by the Ethics Committee of SJHP, China Medical University (Approval No. 2025PS1822K; Filing No. MR-21-25-090420) and conducted in accordance with the principles of the Declaration of Helsinki. Written informed consent was obtained from all adult patients, and consent for minor patients (≤18 years old) was provided by their legal guardians. No additional follow-up or prospective data collection was conducted after ethics approval. Missing baseline laboratory values were handled using median imputation. No patients were excluded because of missing baseline laboratory data.

### 2.2. Outcome Measures and Follow-Up

All predictors were defined as baseline measurements at diagnosis, obtained before any treatment, including neoadjuvant chemotherapy and surgery, and anchored to the date of biopsy-confirmed diagnosis. Peripheral venous blood samples were collected at baseline. ALP and LDH were measured as serum biochemical markers, while FIB and D-dimer were plasma coagulation markers. Laboratory testing was performed in the Department of Laboratory Medicine using standardized procedures, with the BC-6800Plus analyzer (Mindray, Shenzhen, China) used for hematological testing. D-dimer was reported in μg/L DDU without conversion to FEU. The Ki-67 index was assessed by immunohistochemistry on pretreatment biopsy specimens and expressed as the percentage of positively stained tumor cell nuclei. Tumor size was defined as the maximum diameter on baseline CT or MRI in centimeters. Lung metastasis was evaluated on baseline high-resolution chest CT and independently confirmed by two radiologists.

Overall survival was defined as the interval from biopsy-confirmed pathological diagnosis to death from any cause. Death from any cause was considered an OS event, whereas patients alive at the data cutoff were right-censored at their last confirmed date alive. 1-, 3-, and 5-year OS rates were prespecified summary estimates. Secondary endpoints included postoperative functional outcomes and health-related quality of life, assessed at 1, 3, 6, and 12 months after surgery using the Musculoskeletal Tumor Society Score (MSTS-93), Toronto Extremity Salvage Score (TESS), and EQ-5D-5L. EQ-5D-5L health states were converted into utility scores using the Chinese mainland value set developed by Luo et al. [20]. Follow-up was conducted every 3 months through outpatient visits, telephone interviews, and imaging examinations (chest CT and local MRI/CT), with follow-up ending in February 2026. Vital status was determined from outpatient records and telephone follow-up; imaging was used for disease assessment but not as independent evidence of vital status. When direct contact was unsuccessful, censoring was assigned at the last documented date alive.

Postoperative complications were defined as adverse events related to surgery, reconstruction, or fixation that occurred during follow-up after LSS. Local recurrence was recorded separately as an oncological outcome. Wound complications included delayed or poor healing requiring intervention, while mechanical complications included prosthetic or fixation failure and fractures. Venous thromboembolism was recorded when objectively diagnosed. For each complication, the number of patients, events, timing, treatment, and outcomes were recorded when available.

### 2.3. Treatment Plan

LSS was performed when complete tumor resection with an oncologically acceptable margin was considered feasible while preserving or reconstructing major neurovascular structures and maintaining adequate limb function. Reconstruction was performed using prosthetic or biological techniques according to tumor location, extent of resection, skeletal maturity, and local soft-tissue conditions. Expandable prostheses were used when appropriate in skeletally immature patients. All patients achieved negative margins. Because reconstruction type and surgical margin were determined intraoperatively, they were unavailable at the diagnosis-stage prediction time point and were not included as baseline predictors.

Chemotherapy Regimen: All patients completed the same planned neoadjuvant chemotherapy prior to surgery, consisting of two cycles of intravenous chemotherapy with doxorubicin, cisplatin, and methotrexate. The specific regimen is as follows: methotrexate 8–12 g/m^2^, on days 1 and 8; cisplatin 90–120 mg/m^2^, on day 15; and doxorubicin 60 mg/m^2^ on day 17. The interval between the first and second cycles is 14 days. Quantitative histological necrosis rates were not consistently available for retrospective analysis and were therefore not included as candidate predictors. None of the patients received targeted systemic therapy, including anlotinib, during the study period. Subsequent systemic treatment for patients with pulmonary metastases was determined according to clinical course and treatment response and, when applicable, recorded separately from the standardized neoadjuvant regimen.

### 2.4. Construction of the Prognostic Model

Candidate predictors were prespecified based on their availability at the diagnosis-stage prediction time point and prior evidence of prognostic relevance. Nine baseline variables were considered: ALP, D-dimer, LDH, FIB, Ki-67, age, sex, tumor size, and lung metastasis status. Continuous predictors were dichotomized using the cohort-specific Youden index cutoffs described in Section 2.6. All nine candidate predictors were then entered into an L1-regularized Cox proportional hazards (LASSO-Cox) model for simultaneous variable selection and coefficient shrinkage [21].

The LASSO-Cox model was fitted using the glmnet package in R (version 4.5.1), with alpha = 1. The regularization parameter λ was selected by 10-fold cross-validation using cv.glmnet(), with the λ_min criterion [22]. Univariate Cox regression was performed separately to describe unadjusted associations and was not used for predictor screening or model entry [23]. A conventional multivariable Cox model including the LASSO-selected variables was additionally fitted to provide descriptive estimates of hazard ratios and 95% confidence intervals. These estimates were not used as coefficients in the final penalized prediction model.

Given the retrospective nature of the study, the sample size was determined by the number of eligible patients available during the study period. Sample size adequacy was subsequently assessed using the Riley et al. framework implemented in the pmsampsize package (version 1.1.3) [24]. Because no externally justified Cox-Snell R^2^ estimate was available, calculations were performed across assumed R^2^ values of 0.05, 0.10, and 0.15, with a target global shrinkage factor of 0.90, a maximum absolute difference of 0.05 between apparent and adjusted Nagelkerke R^2^, and a margin of error of 0.05 for the estimated 5-year outcome risk. These calculations were based on the nine candidate predictor parameters and did not account for the additional information used in data-derived cutpoint selection or cross-validated tuning of λ.

### 2.5. Model Validation and Performance Evaluation

Internal validation was performed using 1000 bootstrap resamples generated with replacement from the development cohort. The complete model-development procedure, including LASSO-Cox fitting and λ selection, was repeated within each bootstrap resample. Model performance was evaluated in both the bootstrap sample and the original cohort, and optimism was estimated as the difference between these performance measures. Optimism-corrected performance was obtained by subtracting the mean optimism from the apparent performance in the original cohort [25]. Selection frequencies for all nine candidate predictors were also recorded across the bootstrap resamples as an exploratory assessment of variable-selection stability. Bootstrap resampling was used solely for internal validation and was not considered independent external validation [26].

Model discrimination was assessed using Harrell’s C-index and time-dependent area under the receiver operating characteristic curve (AUC) at 12, 24, 36, 48, and 60 months. Harrell’s C-index assessed overall concordance between predicted risk and observed survival outcomes, whereas time-dependent AUC quantified discrimination at specific prediction horizons while accounting for right censoring [27].

Calibration was evaluated at the same prediction horizons using calibration-in-the-large (CITL), calibration slope, and calibration plots. Overall prediction error was assessed using the inverse probability of censoring weighting (IPCW) Brier score. Definitions and interpretations of the discrimination and prediction-error measures used in this study are provided in Supplementary Table S4.

Because the Brier score reflects both discrimination and calibration, it was interpreted as an overall prediction-error measure rather than a standalone measure of calibration. Given the limited number of outcome events and the small number of patients remaining at risk at later prediction horizons, particularly at 60 months, these estimates were interpreted cautiously [28].

Decision curve analysis (DCA) was performed to explore the net benefit of the model for predicting 5-year all-cause mortality across threshold probabilities of 0.10–0.50. Model net benefit was compared with treat-all and treat-none strategies. Because no treatment or surveillance strategy was prospectively linked to the predicted risks, DCA was considered exploratory and was not interpreted as evidence of clinical utility or as a basis for treatment decisions [29].

At the 60-month prediction horizon, time-dependent sensitivity, specificity, positive predictive value (PPV), and negative predictive value (NPV) were estimated using IPCW to account for right censoring. The Youden index (sensitivity + specificity − 1) was used to identify the risk threshold that maximized combined sensitivity and specificity. These threshold-dependent measures were considered exploratory because the threshold was derived and evaluated within the same cohort. Definitions of the calibration, classification, and proportional hazards assessment measures are provided in Supplementary Table S5.

### 2.6. Statistical Analysis

Categorical variables were summarized as frequencies and percentages, whereas continuous variables were summarized using appropriate measures of central tendency and dispersion according to their distributions. Statistical analyses were performed using R software (version 4.5.1), and graphical presentations were generated using R and GraphPad Prism (version 10.5.0). Receiver operating characteristic (ROC) curve analysis for 5-year OS was used to derive cohort-specific cutoffs for ALP, LDH, FIB, D-dimer, tumor size, age, and Ki-67 using the Youden index. These cutoffs were used to dichotomize continuous variables for exploratory group-based analyses and LASSO-Cox modeling. Because the cutoffs were derived and evaluated within the same cohort, they were considered exploratory and were not interpreted as validated clinical thresholds.

Associations between categorical clinicopathological characteristics and dichotomized biomarker levels were assessed using the χ^2^ test. Overall survival was estimated using the Kaplan–Meier method, and survival distributions between groups were compared using the log-rank test. Univariable Cox proportional hazards regression was used to estimate unadjusted associations with OS, with hazard ratios (HRs) and corresponding 95% confidence intervals (CIs) reported. All statistical tests were two-sided, and *p* < 0.05 was considered statistically significant.

### 2.7. AI-Assisted Language Editing

During the preparation of this manuscript, the authors used GPT-5.5 solely for language editing to improve grammatical accuracy, clarity, readability, and linguistic quality. AI-based tools were not used for study conception, data acquisition, statistical analysis, interpretation of results, or formulation of conclusions. The authors remain fully responsible for the content and accuracy of the manuscript.

## 3. Results

### 3.1. Patient Characteristics and Survival Outcomes

Between January 2019 and January 2025, a total of 118 patients were diagnosed with osteosarcoma at our institution. Among these, 92 patients underwent LSS. After applying the predefined inclusion and exclusion criteria, 11 patients were excluded due to loss to follow-up, leaving 81 patients eligible for the final analysis. Baseline clinicopathological characteristics stratified by ALP, LDH, FIB, and D-dimer levels are summarized in Table 1. Representative imaging and intraoperative findings of distal femoral osteosarcoma, including segmental resection and tumor-type prosthetic replacement, are presented in Supplementary Figure S1.

**Figure 2:**
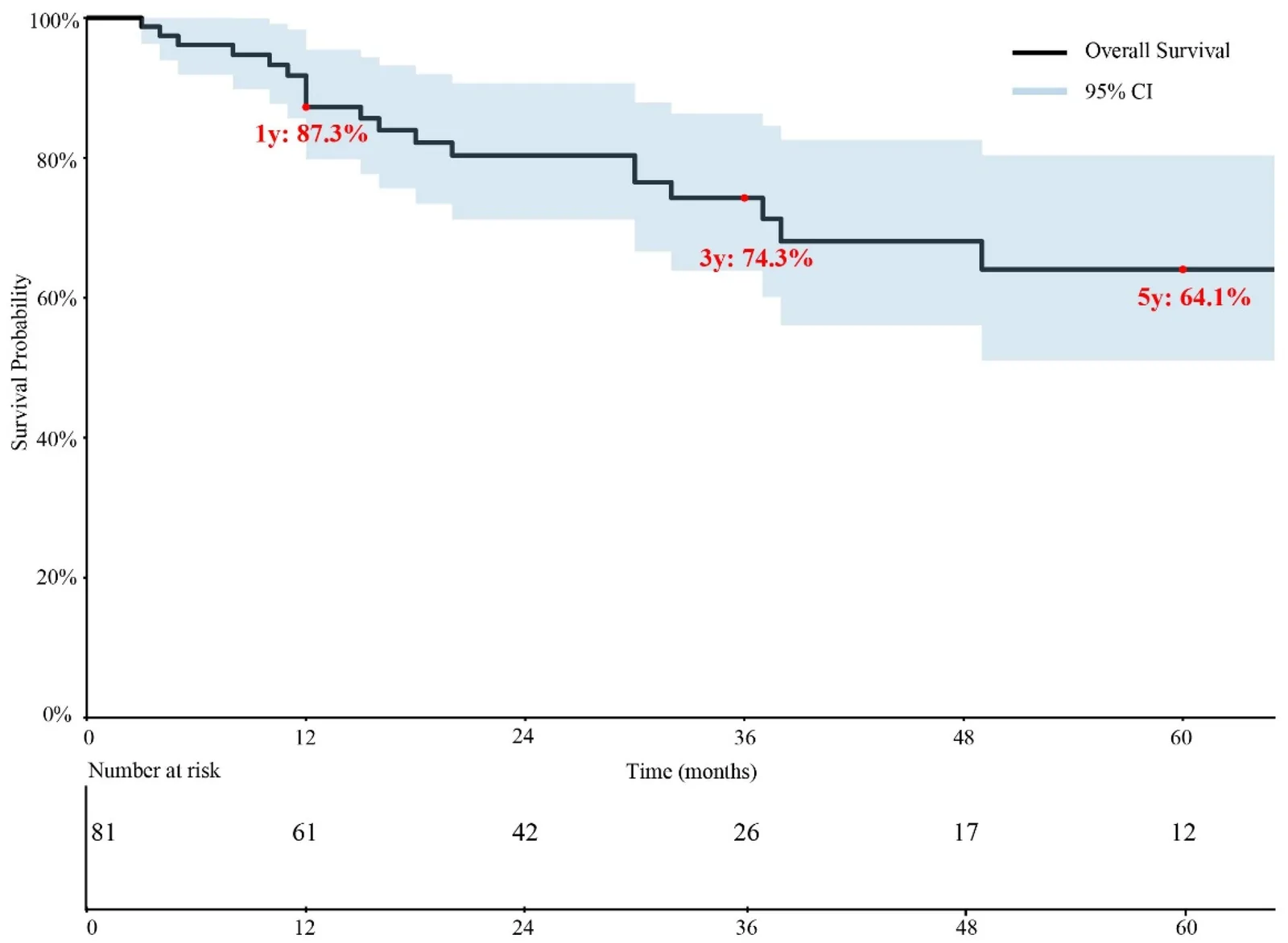
Survival outcomes following LSS in osteosarcoma patients. OS Kaplan–Meier curve for the entire cohort (*n* = 81). The solid line represents the estimated OS probability, with the shaded area indicating the 95% confidence interval. The 1-, 3-, and 5-year OS rates were 87.3% (95%CI: 79.8–95.4%), 74.3% (95%CI: 63.9–86.3%), and 64.1% (95%CI: 51.1–80.4%), respectively.

At the last follow-up, Kaplan–Meier survival analysis demonstrated 1-, 3-, and 5-year OS rates of 87.3%, 74.3%, and 64.1%, respectively, as shown in Figure 2. During follow-up, 62 patients (76.5%) remained alive at last follow-up, whereas 19 patients (23.5%) died due to tumor progression. Functional outcomes improved substantially postoperatively. Longitudinal trajectories of functional and quality-of-life measures are presented in Supplementary Figure S2. However, because the number of evaluable patients decreased over time, especially at later follow-up, these trajectories were considered exploratory and were not interpreted as evidence of sustained improvement for the entire cohort.

### 3.2. Exploratory ROC-Derived Cutoffs for Candidate Predictors

**Figure 3:**
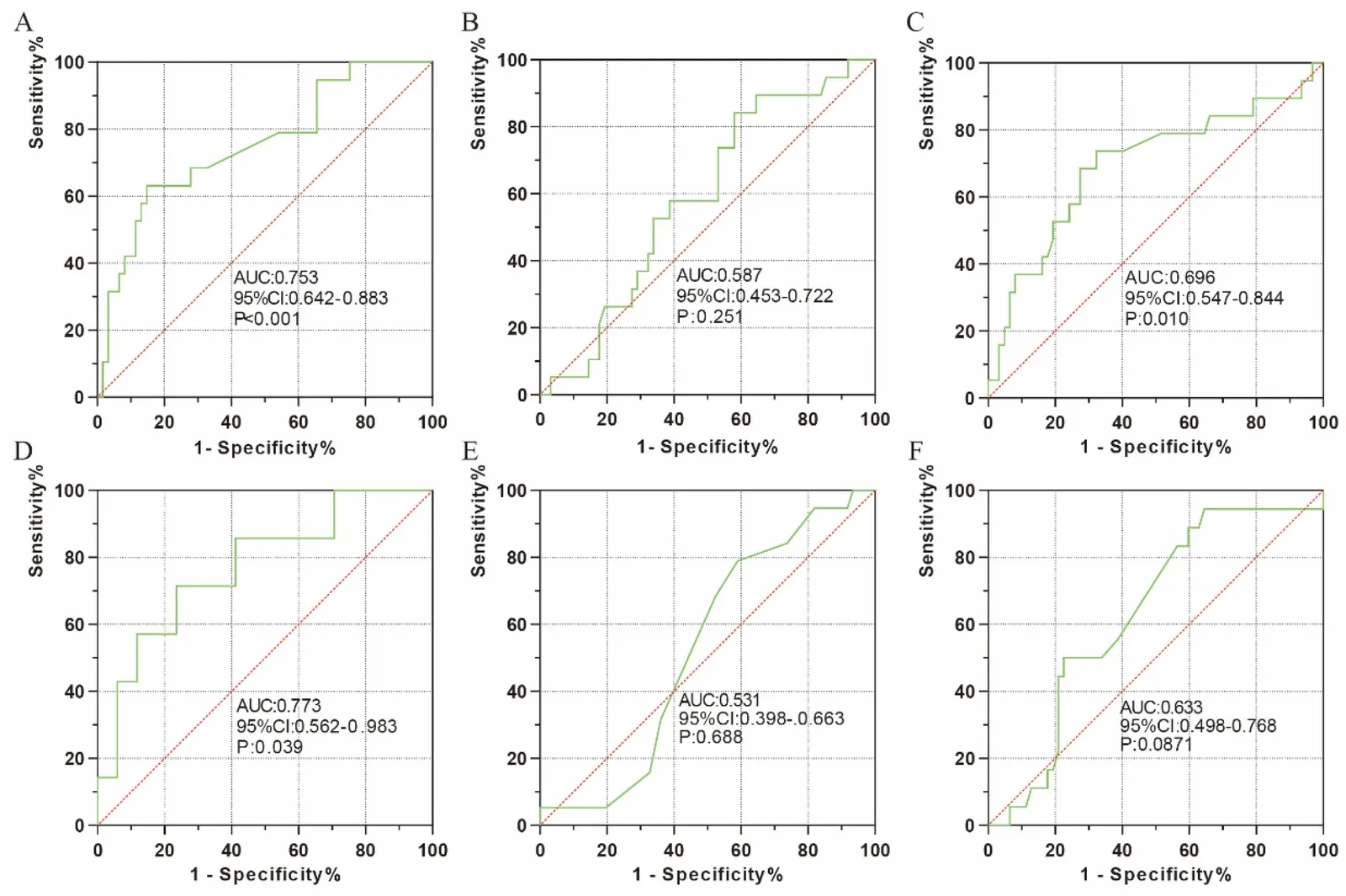
ROC curves for individual prognostic factors predicting overall survival in osteosarcoma patients after LSS. The figure presents the discriminatory performance of six individual candidate predictors: (**A**) ALP: AUC = 0.753 (*p* < 0.001, 95% CI: 0.642–0.883). (**B**) D-dimer: AUC = 0.587 (*p* = 0.251, 95% CI: 0.453–0.722). (**C**) FIB: AUC = 0.696 (*p* = 0.010, 95% CI: 0.547–0.844). (**D**) LDH: AUC = 0.773 (*p* = 0.039, 95% CI: 0.562–0.983). (**E**) Ki-67 index: AUC = 0.531 (*p* = 0.688, 95% CI: 0.398–0.663). (**F**) Tumor size: AUC = 0.633 (*p* = 0.087, 95% CI: 0.498–0.768). ALP, FIB, and LDH reached *p* < 0.05 in this ROC analysis, whereas D-dimer (AUC = 0.587, *p* = 0.251), Ki-67 (AUC = 0.531, *p* = 0.688), and tumor size (AUC = 0.633, *p* = 0.087) did not. The green solid curves represent the ROC curves, whereas the red dashed diagonal line represents the reference line for a non-discriminatory classifier (AUC = 0.50).

ROC curve analysis was performed to derive cohort-specific cutoff values for exploratory risk stratification. The cohort-specific cutoff values selected using the Youden index were 233.1 U/L for ALP, 200 U/L for LDH, 3.02 g/L for FIB, and 113 μg/L (DDU) for D-dimer. The corresponding AUC values were 0.753 (95% CI: 0.642–0.883), 0.773 (95% CI: 0.562–0.883), 0.696 (95% CI: 0.547–0.844), and 0.587 (95% CI: 0.453–0.722), respectively. Notably, D-dimer showed limited individual discrimination (AUC = 0.587, *p* = 0.251); therefore, its ROC/Youden-derived cutoff should be interpreted as an exploratory cohort-specific threshold rather than evidence of robust discrimination. The sensitivities were 0.632, 0.714, 0.737, and 0.842, whereas the specificities were 0.852, 0.765, 0.677, and 0.419, respectively. Based on these exploratory cutoff values, patients were classified into high- and low-expression groups for the reported survival analysis (Figure 3).

### 3.3. Kaplan–Meier Analysis of OS

**Figure 4:**
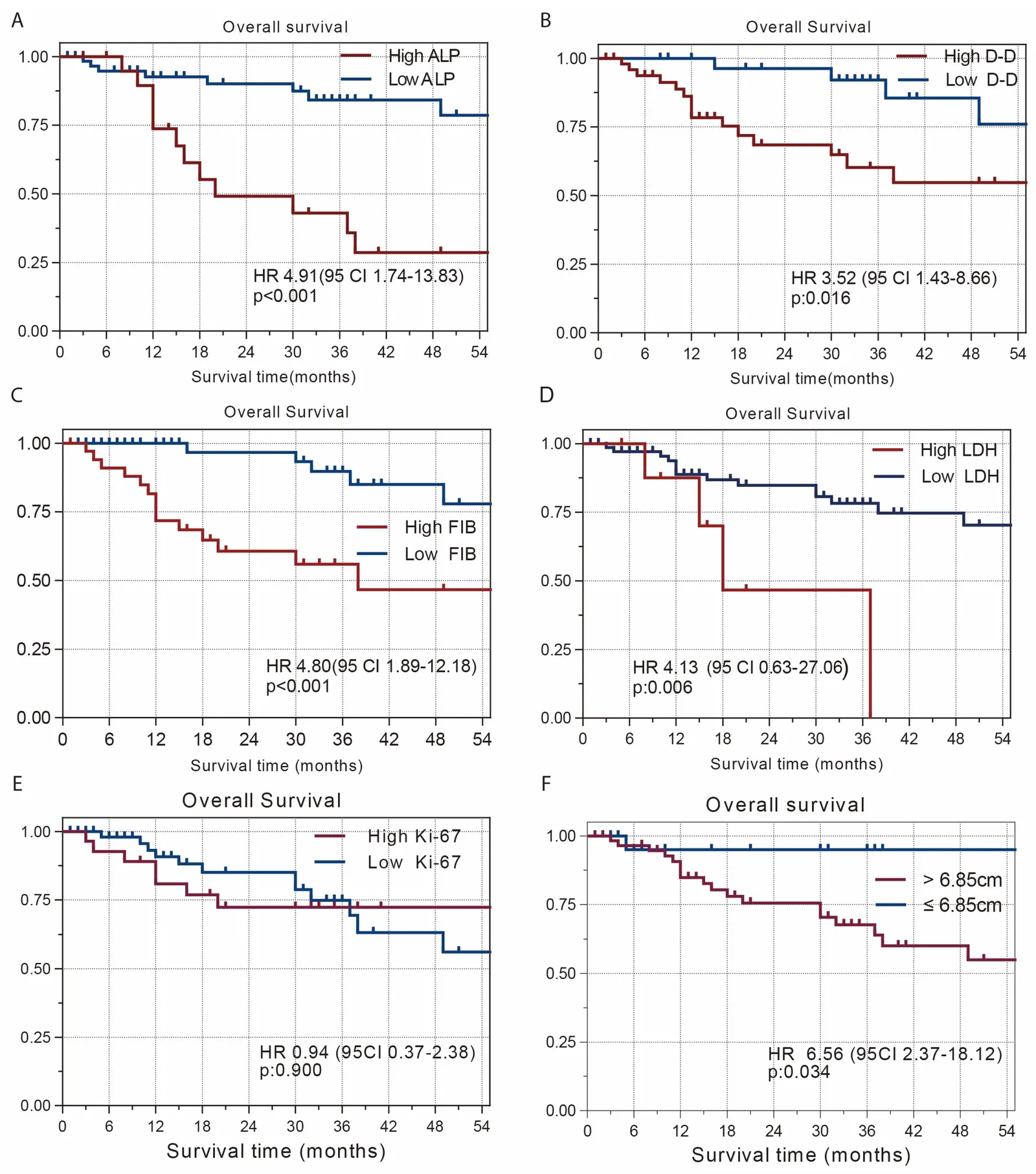
Kaplan–Meier survival curves for individual prognostic factors in osteosarcoma patients after LSS. All *p* values were calculated using the log-rank test: (**A**) OS stratified by baseline ALP levels. Patients with high ALP (>233.1 U/L) had significantly worse OS (*p* < 0.001). (**B**) OS stratified by baseline D-dimer level (>113 vs. ≤113 μg/L DDU; log-rank *p* = 0.016). (**C**) OS stratified by baseline FIB levels. High FIB (>3.02 g/L) was associated with significantly shorter OS (*p* < 0.001). (**D**) OS stratified by baseline lactate dehydrogenase (LDH) levels. High LDH (>200 U/L) predicted significantly poorer survival (*p* = 0.006). (**E**) OS stratified by Ki-67 index. No significant association was found between Ki-67 expression and OS (HR 0.94, 95% CI 0.37–2.38, *p* = 0.900). (**F**) OS stratified by tumor size. Larger tumor size (>6.85 cm) was significantly associated with reduced OS (*p* = 0.016).

Kaplan–Meier analysis showed significantly poorer OS in patients with elevated baseline ALP (*p* < 0.001), LDH (*p* = 0.006), FIB (*p* < 0.001), and D-dimer (*p* = 0.016). The corresponding 5-year OS rates in the high- versus low-level groups were 28.7% versus 78.6% for ALP, 0% versus 77.3% for LDH, 46.7% versus 77.9% for FIB, and 54.8% versus 76.0% for D-dimer. Larger tumor size was also associated with poorer OS (*p* = 0.016), whereas Ki-67 was not significantly associated with OS (Figure 4). Given the cohort-derived cutoffs, these subgroup analyses should be considered exploratory.

### 3.4. Univariable Associations with Overall Survival

Univariable Cox proportional hazards regression was performed to characterize the unadjusted associations between each candidate predictor and OS. Elevated baseline ALP, LDH, FIB, and D-dimer were significantly associated with poorer OS. Tumor size and lung metastasis showed nonsignificant trends toward poorer OS, whereas Ki-67, age, and sex were not significantly associated with OS (Table 2).

### 3.5. LASSO-Cox Model Development and Multivariable Analysis

**Figure 5:**
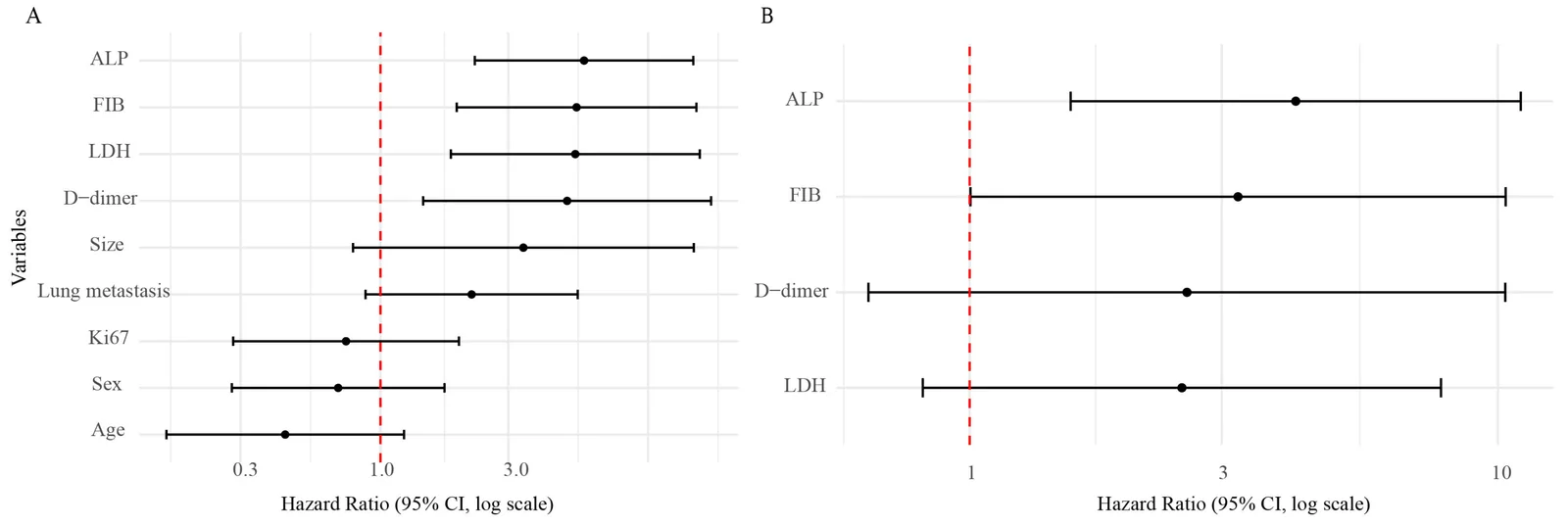
Forest plots of univariate and multivariate Cox proportional hazards regression analyses for OS. (**A**) Univariate analysis displays the HR and 95% CI for each candidate variable when analyzed individually. Elevated baseline levels of ALP, LDH, FIB, and D-dimer were significantly associated with worse overall survival (*p* ≤ 0.05). (**B**) Multivariate analysis shows the HR and 95% CI for the final prognostic variables selected and adjusted in the same model. Consistent with the main results, high baseline ALP (HR = 4.143, 95% CI: 1.553–11.050, *p* = 0.001) and high baseline FIB (HR = 3.220, 95%CI: 1.003–10.345, *p* = 0.050) were identified as independent risk factors for shorter survival. The solid vertical line at HR = 1.0 indicates no effect.

All nine prespecified candidate predictors were entered into the LASSO-Cox model. At λ_min, four variables—ALP, LDH, FIB, and D-dimer—had non-zero coefficients and were retained in the penalized prediction model. Details of predictor selection, LASSO coefficients, and proportional hazards assessment are provided in Supplementary Table S2. The final model specification, including the retained predictors and corresponding penalized coefficients, is provided in Supplementary Table S3. A conventional multivariable Cox model including these LASSO-selected variables was additionally fitted for descriptive estimation of adjusted associations with OS. In this analysis, elevated ALP remained significantly associated with poorer OS (HR = 4.143, 95% CI: 1.553–11.050, *p* = 0.001), while FIB showed a borderline association (HR = 3.220, 95% CI: 1.003–10.345, *p* = 0.050). LDH and D-dimer were not statistically significant after mutual adjustment (Figure 5).

### 3.6. Model Performance and Internal Validation

The LASSO-Cox model showed an apparent Harrell’s C-index of 0.838. In Bootstrap internal validation, the mean C-index was 0.828. After correction for optimism, the C-index decreased to 0.780 (95% CI: 0.653–0.846). The apparent time-dependent AUCs at 12, 24, 36, 48, and 60 months were 0.831, 0.948, 0.864, 0.923, and 0.871, respectively. The corresponding mean AUCs across bootstrap resamples were 0.832, 0.935, 0.858, 0.906, and 0.854 at 12, 24, 36, 48, and 60 months, respectively. After subtraction of the mean bootstrap optimism, the optimism-corrected AUCs were 0.771 (95% CI: 0.614–0.898), 0.908 (95% CI: 0.794–0.974), 0.810 (95% CI: 0.685–0.944), 0.866 (95% CI: 0.738–0.975), and 0.814 (95% CI: 0.707–0.930), respectively. Thus, the definitive optimism-corrected AUCs for 1-, 3-, and 5-year OS were 0.771, 0.810, and 0.814, respectively.

Calibration-in-the-large estimates at 12, 24, 36, 48, and 60 months were 0.059, 0.009, −0.028, −0.052, and −0.065, respectively, while the corresponding calibration slopes were 0.268, 0.633, 0.699, 0.947, and 0.897. The optimism-corrected IPCW Brier scores at the same prediction horizons were 0.139, 0.127, 0.188, 0.193, and 0.237, respectively. Uncertainty in calibration was substantial at some prediction horizons, and only 12 patients remained at risk at 60 months; therefore, estimates at later horizons should be interpreted cautiously.

Across 1000 bootstrap resamples, selection frequencies were 0.99 for FIB, 0.94 for ALP, 0.92 for D-dimer, and 0.80 for LDH. Selection frequencies for pulmonary metastasis, sex, age, Ki-67, and tumor size were 0.64, 0.56, 0.41, 0.40, and 0.40, respectively.

Schoenfeld residual testing showed no statistically significant evidence of proportional hazards violation for individual predictors (ALP, *p* = 0.241; LDH, *p* = 0.062; FIB, *p* = 0.074; D-dimer, *p* = 0.071). However, the global test was significant (*p* = 0.044), indicating potential departure from the proportional hazards assumption at the model level (Supplementary Table S1; Supplementary Figure S3).

**Figure 6:**
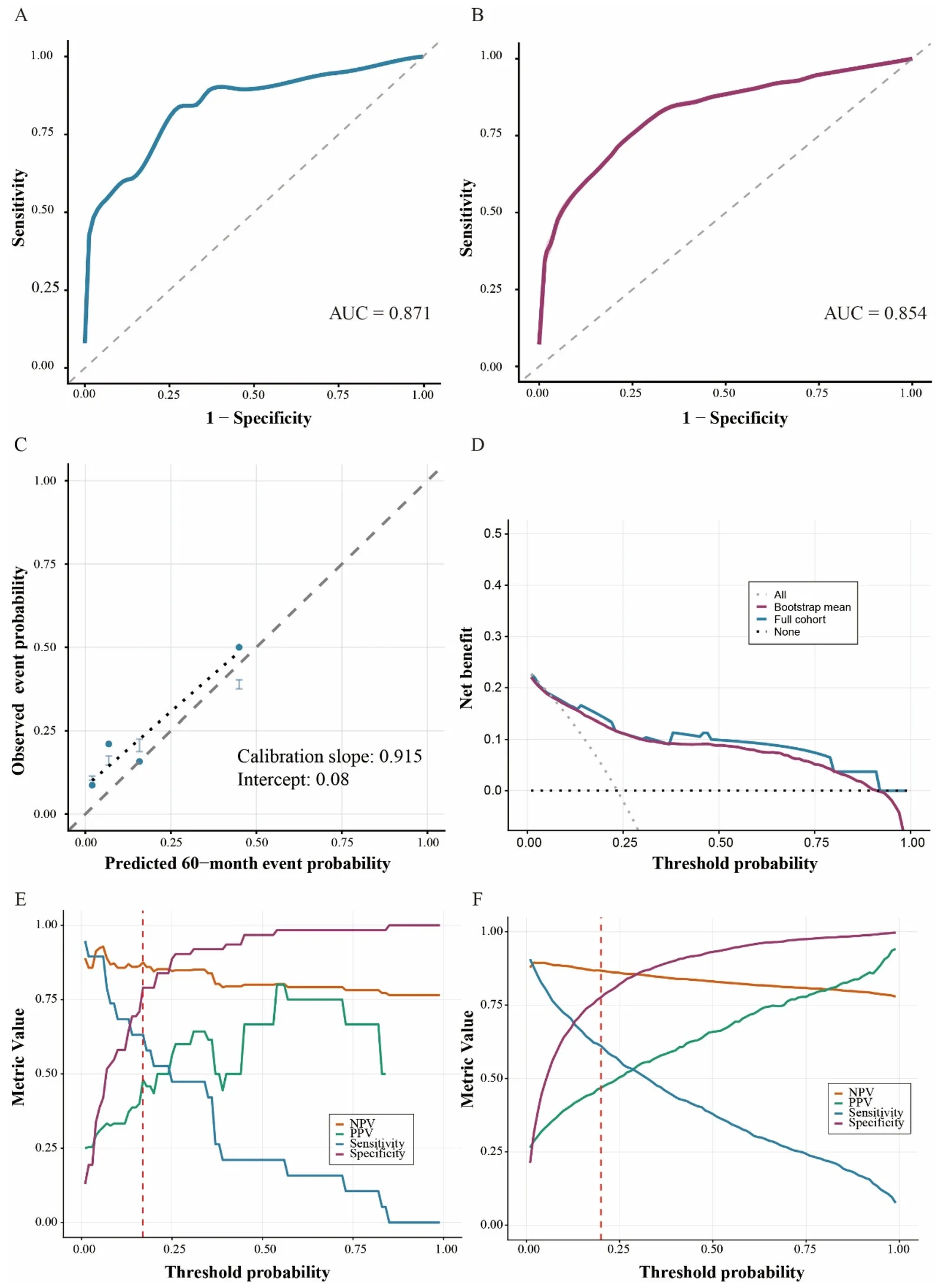
Internal performance evaluation of the prediction model: (**A**) Apparent ROC curve for the full dataset at 60 months (AUC = 0.871); the diagonal dashed line represents a non-informative classifier (AUC = 0.50). (**B**) Mean ROC curve across bootstrap resamples at 60 months (AUC = 0.854). (**C**) Calibration curve at 60 months; the solid diagonal line indicates perfect calibration, and the dashed line represents the calibration obtained from the bootstrap validation analysis (calibration slope = 0.915, intercept = 0.08). (**D**) DCA showing positive net benefit across the reported threshold probabilities in the full dataset (blue) and Bootstrap internal validation (orange). (**E**) Performance curves in the full dataset; the threshold of 0.17 yielded a Youden index of 0.422. (**F**) Performance curves after Bootstrap internal validation; the threshold of 0.20 yielded a Youden index of 0.388. These panels report apparent and internally corrected performance from the same single-center dataset and should not be interpreted as evidence of external robustness or clinical utility.

Decision curve analysis showed greater net benefit for the model than for the treat-all and treat-none strategies across part of the prespecified threshold-probability range of 0.10–0.50 (Figure 6). Because no clinical intervention was prospectively linked to the predicted risk thresholds, this analysis was considered exploratory. Post hoc sample size assessment indicated that the available cohort was smaller than the estimated requirements across assumed Cox–Snell R^2^ values of 0.05–0.15 (estimated sample sizes, 494–1575 patients; estimated outcome events, 116–370), further supporting cautious interpretation of the model-development results.

### 3.7. Postoperative Complications and Oncological Events

During postoperative follow-up, 14 of 81 patients (17.3%) experienced at least one surgery-related complication. The most common complications were periprosthetic fracture or internal fixation failure (7/81, 8.6%), followed by poor or delayed wound healing (2/81, 2.5%), prosthesis fracture (1/81, 1.2%), and deep vein thrombosis (1/81, 1.2%). Most complications were managed conservatively or by revision surgery, and no complication-related deaths occurred.

Four patients (4.9%) subsequently underwent amputation because of uncontrolled infection or local tumor progression after the initial LSS. Local recurrence, analyzed separately as an oncological outcome rather than a surgical complication, occurred in four patients (4.9%).

## 4. Discussion

Osteosarcoma is a highly complex and heterogeneous malignant tumor. Comprehensive treatment strategies centered on limb salvage have become the standard therapeutic approach; however, substantial interindividual variability remains in postoperative recurrence and disease progression risk [30]. In this single-center retrospective cohort of patients with osteosarcoma undergoing limb-salvage surgery, higher baseline levels of ALP, LDH, FIB, and D-dimer were associated with poorer OS in exploratory survival analyses. LASSO-Cox modeling retained all four biomarkers in the penalized prediction model, although their roles differed in conventional multivariable Cox analysis: ALP remained significantly associated with OS, FIB showed a borderline association, whereas LDH and D-dimer were not statistically significant after mutual adjustment. After bootstrap correction for optimism, the model showed a C-index of 0.780 and time-dependent AUCs of 0.771, 0.810, and 0.814 for 1-, 3-, and 5-year OS, respectively. These findings suggest that routinely available baseline laboratory biomarkers may contain complementary prognostic information; however, given the limited number of outcome events and lack of external validation, the model should be regarded as exploratory.

The prognostic associations of these biomarkers are biologically plausible because they reflect distinct aspects of osteosarcoma biology and host response. ALP is closely linked to osteoblastic activity and bone turnover and has long been associated with tumor burden and prognosis in osteosarcoma [31,32,33]. LDH reflects glycolytic activity and tissue turnover; elevated LDH may accompany aggressive tumor metabolism, lactate accumulation, and microenvironmental changes associated with tumor progression [34,35,36,37]. In parallel, FIB and D-dimer reflect activation of coagulation and fibrinolysis, processes increasingly recognized as components of cancer progression and metastasis [38,39,40,41,42,43,44,45,46,47]. Fibrin-rich microenvironments may facilitate tumor-cell survival and dissemination, whereas elevated D-dimer may reflect increased fibrin formation and degradation. Taken together, these markers may capture complementary dimensions of tumor burden, metabolic activity, and systemic coagulation responses rather than representing interchangeable measures of the same biological process.

Not all candidate predictors demonstrated consistent prognostic value. Ki-67 was not significantly associated with OS in the present cohort, despite its reported prognostic relevance in some previous studies [48,49,50]. This discrepancy may reflect intratumoral heterogeneity, limited representation of biopsy specimens, differences in cutoff definitions, and the limited statistical power of the present cohort. Accordingly, the absence of a significant association should not be interpreted as evidence that Ki-67 lacks prognostic relevance in osteosarcoma.

Our findings are broadly consistent with previous reports supporting the prognostic relevance of circulating biochemical and coagulation markers in osteosarcoma. Pu et al., for example, reported prognostic associations for preoperative D-dimer and fibrinogen, although their cohort, endpoint definition, and modeling strategy differed from those of the present study [51]. A distinguishing feature of our approach was the restriction of predictors to routinely available baseline laboratory measurements obtained before treatment and their integration within a penalized survival model. This design prioritizes accessibility and reproducibility; however, it also addresses a narrower prognostic question than models incorporating tumor stage, imaging, histological response, or treatment-related variables. The present model should therefore be considered complementary to, rather than a replacement for, established clinicopathological prognostic assessment.

An important distinction should be made between variable retention in the LASSO-Cox model and statistical significance in conventional multivariable Cox regression. LASSO selects and shrinks coefficients according to their contribution to penalized predictive performance rather than conventional hypothesis-testing criteria. Therefore, retention of LDH and D-dimer despite nonsignificant adjusted Cox coefficients is not inherently contradictory. Nevertheless, the stability of predictor selection remains uncertain in a cohort with only 19 deaths. Although bootstrap selection frequencies were relatively high for FIB, ALP, D-dimer, and LDH, these findings require confirmation in independent data. The reduction in the C-index from 0.838 to 0.780 after optimism correction illustrates the importance of accounting for overfitting. Similarly, optimism-corrected time-dependent AUCs remained above 0.77 at the evaluated 1-, 3-, and 5-year horizons, but these estimates should not be interpreted as evidence of clinical utility. Calibration estimates were imprecise at several horizons, and the global Schoenfeld residual test suggested possible departure from the proportional hazards assumption. These findings further support cautious interpretation and external validation before clinical application.

Postoperative functional and health-related quality-of-life measures showed improvement during follow-up; however, these findings should be interpreted separately from the survival prediction model. Functional recovery after major musculoskeletal surgery is multifactorial and may be influenced by rehabilitation exposure, physical function, pain, complications, treatment-related factors, and patient participation [52,53,54,55]. Moreover, the decreasing number of evaluable patients at later follow-up introduces the possibility of survivor and limb-retention bias. The functional findings should therefore be considered descriptive and hypothesis-generating rather than evidence of a sustained treatment effect.

Several limitations should be acknowledged. First, this was a single-center retrospective study with only 81 patients and 19 deaths. Post hoc sample size assessment indicated that the available outcome information was insufficient for stable model development under the examined assumptions; therefore, predictor selection and performance estimates may remain unstable despite penalization and bootstrap optimism correction. Second, continuous predictors were dichotomized using cohort-derived ROC/Youden cutoffs. This approach discards information, may reduce statistical efficiency, and introduces additional optimism because cutoffs were selected and evaluated within the same cohort. This issue is particularly relevant for ALP because its physiological distribution varies substantially with age and skeletal maturity. Third, the median follow-up was 34 months, and only 12 patients remained at risk at 60 months, resulting in considerable uncertainty in 5-year performance estimates. Fourth, the biomarker-focused model did not incorporate several established clinicopathological and treatment-related predictors, including detailed stage, histological response, treatment completion, and subsequent treatment modifications; consequently, its incremental value beyond conventional prognostic information could not be determined. Fifth, baseline laboratory biomarkers may be influenced by non-tumor conditions, and dynamic changes during treatment were not evaluated. Finally, the model lacks independent external validation. Future studies should use larger multicenter cohorts, retain continuous predictors where appropriate, investigate nonlinear effects, incorporate prespecified clinical predictors, and evaluate incremental performance and clinical utility through external and prospective validation.

## 5. Conclusions

Routine baseline laboratory biomarkers were associated with OS in patients with osteosarcoma undergoing limb-salvage surgery, and their integration in a LASSO-Cox model showed prognostic discrimination after bootstrap correction for optimism. However, given the limited number of outcome events and the absence of external validation, the model should be considered exploratory rather than ready for clinical implementation. Larger multicenter studies with independent external validation are needed to confirm the reproducibility of these findings and determine whether these biomarkers provide incremental prognostic value beyond established clinicopathological predictors.

## Figures and Tables

**Table 1:** Characteristics of the included patients.

Variable	Number (*n* = 81)
Age, years	
>18, *n* (%)	50 (61.7)
≤18, *n* (%)	31 (38.3)
Sex	
Female, *n* (%)	43 (53.1)
Male, *n* (%)	38 (46.9)
Location of primary tumor	
Femur, *n* (%)	48 (59.3)
Tibia, *n* (%)	16 (19.8)
Humerus, *n* (%)	5 (6.2)
Fibula, *n* (%)	4 (4.9)
Pelvis, *n* (%)	5 (6.2)
Clavicle, *n* (%)	1 (1.2)
Rib, *n* (%)	1 (1.2)
Scapular, *n* (%)	1 (1.2)
Tumor size, cm	
>6.85, *n* (%)	57 (70.4)
≤6.85, *n* (%)	24 (29.6)
Lung metastasis	
Yes, *n* (%)	22 (27.2)
No, *n* (%)	59 (72.8)
Baseline ALP level in U/L	233.1 [43.2, 1833]
Baseline D-dimer level in μg/L (DDU)	113.0 [4, 4870]
Baseline LDH level in U/L	200.0 [108, 577]
Baseline Ki-67 level in %	50 [1, 95]
Baseline FIB level in g/L	3.02 [2.20, 7.93]
Death due to disease progression	
Yes, *n* (%)	19 (23.5)
No, *n* (%)	62 (76.5)
Unknown, *n* (%)	0 (0.0)

Continuous variables are presented as median [minimum, maximum] and categorical variables as n (%).

**Table 2:** Univariable and multivariable Cox proportional hazards regression analyses of overall survival.

Variables	Univariable Analysis		Multivariable Analysis	
	HR (95% CI)	*p* Value	HR (95% CI)	*p* Value
ALP	5.762 (2.251–14.747)	0.001	4.143 (1.553–11.050)	0.001
D-dimer	4.980 (1.443–17.184)	0.011	2.578 (0.643–10.329)	0.181
LDH	5.342 (1.831–15.588)	0.002	2.522 (0.815–7.802)	0.108
FIB	5.400 (1.926–15.145)	0.001	3.220 (1.003–10.345)	0.050
Ki-67	0.743 (0.282–1.962)	0.549		
Age	0.440 (0.159–1.224)	0.116		
Size	3.417 (0.789–14.801)	0.100		
Lung metastasis	2.190 (0.879–5.457)	0.093		
Sex	0.695 (0.278–1.733)	0.435		

## Data Availability

The data presented in this study are not publicly available due to the inclusion of sensitive patient information but are available from the corresponding author upon reasonable request.

## Supplementary Materials

The following supporting information can be downloaded at https://www.mdpi.com/article/10.3390/jcm15186962/s1, Figure S1: Representative imaging and intraoperative images of distal femoral osteosarcoma, demonstrating segmental resection and tumor-type prosthetic replacement; Figure S2: Exploratory longitudinal assessment of postoperative functional status and health-related quality of life; Figure S3: Schoenfeld residual-based assessment of the proportional-hazards assumption for individual biomarkers; Table S1: Proportional hazards assumption test for the final four-biomarker Cox model; Table S2: Predictor selection, LASSO coefficients, and proportional hazards assessment; Table S3: Final LASSO-Cox model specification; Table S4: Definitions of discrimination and prediction error measures; Table S5: Definitions of calibration, classification, and proportional hazards assessment.

## Abbreviations

OS	Overall survival
LSS	Limb-salvage surgery
ALP	Alkaline phosphatase
LDH	Lactate dehydrogenase
FIB	Fibrinogen
C-index	The Harrell’s concordance index
AUC	The area under the receiver operating characteristic curve
ROC curves	Receiver operating characteristic curves
BS	Brier score
DCA	Decision curve analysis
PPV	Positive predictive value
NPV	Negative predictive value
HR	Hazard ratio
CI	Confidence interval
CLSI	Laboratory Standards Institute
CITL	Calibration-in-the-large
IPCW	Inverse probability of censoring weighting
SJHP	Shengjing Hospital of China Medical University

## References

[1] Czarnecka A.M., Synoradzki K., Firlej W., Bartnik E., Sobczuk P., Fiedorowicz M., Grieb P., Rutkowski P. (2020). Molecular Biology of Osteosarcoma. Cancers.

[2] Mirabello L., Troisi R.J., Savage S.A. (2009). Osteosarcoma Incidence and Survival Rates from 1973 to 2004: Data from the Surveillance, Epidemiology, and End Results Program. Cancer.

[3] Ullah F., Altaf W., Khan D., Khan N.A., Akbar S., Muhammad Z. (2025). Comparative Outcomes of Limb Salvage Surgery Versus Amputation in Osteosarcoma: A Five-Year Follow-Up Study From a Tertiary Care Center. Cureus.

[4] He X., Gao Z., Xu H., Zhang Z., Fu P. (2017). A Meta-Analysis of Randomized Control Trials of Surgical Methods with Osteosarcoma Outcomes. J. Orthop. Surg..

[5] Papakonstantinou E., Stamatopoulos A., Athanasiadis D.I., Kenanidis E., Potoupnis M., Haidich A.-B., Tsiridis E. (2020). Limb-Salvage Surgery Offers Better Five-Year Survival Rate than Amputation in Patients with Limb Osteosarcoma Treated with Neoadjuvant Chemotherapy. A Systematic Review and Meta-Analysis. J. Bone Oncol..

[6] Kaneuchi Y., Yoshida S., Fujiwara T., Evans S., Abudu A. (2022). Limb Salvage Surgery Has a Higher Complication Rate than Amputation but Is Still Beneficial for Patients Younger than 10 Years Old with Osteosarcoma of an Extremity. J. Pediatr. Surg..

[7] Farmer S.H., O’Brien A.C., Feustel P.J., DiCaprio M.R. (2026). Time to Treatment Initiation and Overall Survival in Osteosarcoma: A National Cancer Database Analysis. Bone Jt. Open.

[8] Kansara M., Teng M.W., Smyth M.J., Thomas D.M. (2014). Translational Biology of Osteosarcoma. Nat. Rev. Cancer.

[9] Collins G.S., Dhiman P., Andaur Navarro C.L., Ma J., Hooft L., Reitsma J.B., Logullo P., Beam A.L., Peng L., Van Calster B. (2021). Protocol for Development of a Reporting Guideline (TRIPOD-AI) and Risk of Bias Tool (PROBAST-AI) for Diagnostic and Prognostic Prediction Model Studies Based on Artificial Intelligence. BMJ Open.

[10] Sever N., Şimşek F., Onur İ.D., Arvas H., Guliyev T., Şakalar T., Çiçek C.M., Orman S., Çetin E.B., Kayaş K. (2025). Prognostic Factors in High Grade Osteosarcoma Patients Who Received Neoadjuvant Therapy and Subsequently Underwent Surgery: Data from the Turkish Oncology Group. J. Clin. Med..

[11] Soff G. (2019). Thrombosis and Hemostasis in Cancer. Scope of the Problem and Overview. Cancer Treat. Res..

[12] Gu R., Sun Y. (2018). Does Serum Alkaline Phosphatase Level Really Indicate the Prognosis in Patients with Osteosarcoma? A Meta-Analysis. J. Cancer Res. Ther..

[13] Huang Y., Liu B., Sun Y., Zhang J., Yao Y., He A. (2016). The Prognostic Value of D-Dimer Levels in Metastatic Osteosarcoma Patients Treated with Second-Line Chemotherapy. Oncotarget.

[14] Ay C., Dunkler D., Pirker R., Thaler J., Quehenberger P., Wagner O., Zielinski C., Pabinger I. (2012). High D-Dimer Levels Are Associated with Poor Prognosis in Cancer Patients. Haematologica.

[15] Huang Y., Shen Z., Yao Y., He A., Min D. (2021). The Plasma Concentration of D-Dimer Is Associated with Neoadjuvant-Chemotherapy Efficacy and the Prognosis in Osteosarcoma. OncoTargets Ther..

[16] Sahni A., Khorana A.A., Baggs R.B., Peng H., Francis C.W. (2006). FGF-2 Binding to Fibrin(Ogen) Is Required for Augmented Angiogenesis. Blood.

[17] Wang M., Zhang G., Zhang Y., Cui X., Wang S., Gao S., Wang Y., Liu Y., Bae J.H., Yang W.-H. (2020). Fibrinogen Alpha Chain Knockout Promotes Tumor Growth and Metastasis through Integrin–AKT Signaling Pathway in Lung Cancer. Mol. Cancer Res..

[18] Krajewska E., Lewis C.E., Chen Y.-Y., Welford A., Tazzyman S., Staton C.A. (2010). A Novel Fragment Derived from the β Chain of Human Fibrinogen, Β43–63, Is a Potent Inhibitor of Activated Endothelial Cells in Vitro and in Vivo. Br. J. Cancer.

[19] Kim B.-C., Kim J., Kim K., Byun B.H., Lim I., Kong C.-B., Song W.S., Koh J.-S., Woo S.-K. (2021). Preliminary Radiogenomic Evidence for the Prediction of Metastasis and Chemotherapy Response in Pediatric Patients with Osteosarcoma Using 18F-FDF PET/CT, EZRIN and KI67. Cancers.

[20] Luo N., Liu G., Li M., Guan H., Jin X., Rand-Hendriksen K. (2017). Estimating an EQ-5D-5L Value Set for China. Value Health.

[21] Wang Q., Qiao W., Zhang H., Liu B., Li J., Zang C., Mei T., Zheng J., Zhang Y. (2022). Nomogram Established on Account of Lasso-Cox Regression for Predicting Recurrence in Patients with Early-Stage Hepatocellular Carcinoma. Front. Immunol..

[22] Yang L., Qu Q., Hao Z., Sha K., Li Z., Li S. (2022). Powerful Identification of Large Quantitative Trait Loci Using Genome-Wide R/Glmnet-Based Regression. J. Hered..

[23] Friedman J., Hastie T., Tibshirani R. (2010). Regularization Paths for Generalized Linear Models via Coordinate Descent. J. Stat. Softw..

[24] Riley R.D., Snell K.I., Ensor J., Burke D.L., Harrell F.E., Moons K.G., Collins G.S. (2019). Minimum Sample Size for Developing a Multivariable Prediction Model: PART II-Binary and Time-to-event Outcomes. Stat. Med..

[25] Zhou X.H., Higgs R.E. (1998). COMPROC and CHECKNORM: Computer Programs for Comparing Accuracies of Diagnostic Tests Using ROC Curves in the Presence of Verification Bias. Comput. Methods Programs Biomed..

[26] Wang J., Chen T., Fu Y., Zhu T., Feng Y., Feng Y., Zhang X., Cai Y., Gao L., Lin Y. (2025). Development and Preliminary Validation of a Predictive Model for IgA Nephropathy Progression. Sci. Rep..

[27] Wen G., Zeng Y., Sun Y. (2025). A Time-Dependent Model for Quantifying Postoperative Inflammatory Trajectories and Predicting Survival in Early Triple-Negative Breast Cancer. World J. Surg. Oncol..

[28] Alba A.C., Agoritsas T., Walsh M., Hanna S., Iorio A., Devereaux P.J., McGinn T., Guyatt G. (2017). Discrimination and Calibration of Clinical Prediction Models: Users’ Guides to the Medical Literature. JAMA.

[29] Vickers A.J., Elkin E.B. (2006). Decision Curve Analysis: A Novel Method for Evaluating Prediction Models. Med. Decis. Mak. Int. J. Soc. Med. Decis. Mak..

[30] Hobusch G.M., Hofer C., Döring K., Ellersdorfer F., Kelaridis T., Windhager R. (2025). Is Rotationplasty Still a Reasonable Reconstruction Option for Patients With a Femoral Bone Sarcoma? A Comparative Study of Patients with a Minimum of 20 Years of Follow-up After Rotationplasty and Lower Extremity Amputation. Clin. Orthop..

[31] Strauss S.J., Frezza A.M., Abecassis N., Bajpai J., Bauer S., Biagini R., Bielack S., Blay J.Y., Bolle S., Bonvalot S. (2021). Bone Sarcomas: ESMO–EURACAN–GENTURIS–ERN PaedCan Clinical Practice Guideline for Diagnosis, Treatment and Follow-Up. Ann. Oncol..

[32] Chu X., Mi B., Xiong Y., Wang R., Liu T., Hu L., Yan C., Zeng R., Lin J., Fu H. (2025). Bioactive Nanocomposite Hydrogel Enhances Postoperative Immunotherapy and Bone Reconstruction for Osteosarcoma Treatment. Biomaterials.

[33] Mensali N., Köksal H., Joaquina S., Wernhoff P., Casey N.P., Romecin P., Panisello C., Rodriguez R., Vimeux L., Juzeniene A. (2023). ALPL-1 Is a Target for Chimeric Antigen Receptor Therapy in Osteosarcoma. Nat. Commun..

[34] Jiang Y., Li F., Gao B., Ma M., Chen M., Wu Y., Zhang W., Sun Y., Liu S., Shen H. (2021). KDM6B-Mediated Histone Demethylation of LDHA Promotes Lung Metastasis of Osteosarcoma. Theranostics.

[35] Mei Z., Shen Z., Pu J., Liu Q., Liu G., He X., Wang Y., Yue J., Ge S., Li T. (2024). NAT10 Mediated ac4C Acetylation Driven m6A Modification via Involvement of YTHDC1-LDHA/PFKM Regulates Glycolysis and Promotes Osteosarcoma. Cell Commun. Signal..

[36] Xu Y., Yang L., Li M., Shu H., Jia N., Gao Y., Shi R., Yang X., Zhang Z., Zhang L. (2024). Anti-Osteosarcoma Trimodal Synergistic Therapy Using NiFe-LDH and MXene Nanocomposite for Enhanced Biocompatibility and Efficacy. Acta Pharm. Sin. B.

[37] Alissa M., Alghamdi S.A., Safhi A.Y., Binshaya A.S., Juraybi T.N., Alqarni A.M., Albati A.A., Abalkhail A. (2026). Flubendiamide Induces Progressive Myocardial Injury via Targeting VEGF-Angiogenesis: Evidence from Radiological, Biochemical, and Histological Analysis. Tissue Cell.

[38] Lin M., He J., Zhang X., Sun X., Dong W., Zhang R., Xu Y., Lv L. (2023). Targeting Fibrinogen-like Protein 1 Enhances Immunotherapy in Hepatocellular Carcinoma. J. Clin. Investig..

[39] Li L., Lin Y., Liu K., Huang R., Wen W., Huang Y., Liu M., Zhou C., Ding S., Luo B. (2024). Multiple-Effect Combined Hydrogels: “Temporal Regulation” Treatment of Osteosarcoma-Associated Bone Defects with Switchable Hyperthermia and Bioactive Agents. Adv. Healthc. Mater..

[40] Mego M., Karaba M., Minarik G., Benca J., Sedlácková T., Tothova L., Vlkova B., Cierna Z., Janega P., Luha J. (2015). Relationship between Circulating Tumor Cells, Blood Coagulation, and Urokinase-Plasminogen-Activator System in Early Breast Cancer Patients. Breast J..

[41] Franchini M., Focosi D., Pezzo M.P., Mannucci P.M. (2024). How We Manage a High D-Dimer. Haematologica.

[42] van Es N., Takada T., Kraaijpoel N., Klok F.A., Stals M.A.M., Büller H.R., Courtney D.M., Freund Y., Galipienzo J., Le Gal G. (2023). Diagnostic Management of Acute Pulmonary Embolism: A Prediction Model Based on a Patient Data Meta-Analysis. Eur. Heart J..

[43] Rose P.G., Terrien J.M., Baker S. (1994). Plasma D-Dimer and Peritoneal CA-125 Levels as Predictors of Disease Status in Ovarian Carcinoma. J. Surg. Oncol..

[44] Reed G.L., Houng A.K., Wang D. (2014). Microvascular Thrombosis, Fibrinolysis, Ischemic Injury, and Death after Cerebral Thromboembolism Are Affected by Levels of Circulating A2-Antiplasmin. Arterioscler. Thromb. Vasc. Biol..

[45] Singh S., Houng A.K., Wang D., Reed G.L. (2016). Physiologic Variations in Blood Plasminogen Levels Affect Outcomes after Acute Cerebral Thromboembolism in Mice: A Pathophysiologic Role for Microvascular Thrombosis. J. Thromb. Haemost..

[46] Vahid Dastjerdi M., Ahmari S., Alipour S., Tehranian A. (2015). The Comparison of Plasma D-Dimer Levels in Benign and Malignant Tumors of Cervix, Ovary and Uterus. Int. J. Hematol.-Oncol. Stem Cell Res..

[47] Kaplan K.L., Mather T., DeMarco L., Solomon S. (1989). Effect of Fibrin on Endothelial Cell Production of Prostacyclin and Tissue Plasminogen Activator. Arterioscler. Off. J. Am. Heart Assoc. Inc..

[48] Wen Y., Song Z., Li Q., Zhang D., Li X., Yu J., Li Z., Ren X., Zhang J., Liu Q. (2024). Development and Validation of a Model for Predicting the Expression of Ki-67 in Pancreatic Ductal Adenocarcinoma with Radiological Features and Dual-Energy Computed Tomography Quantitative Parameters. Insights Imaging.

[49] Wang Q., Ma Q., Li X., Ben S., Xue J., Shang T., Jing X., Liu A. (2026). Predictive Model of Ki67 Expression Level in Osteosarcoma Based on Weakly Supervised Segmentation and Multi-Type Feature Fusion. Comput. Methods Programs Biomed..

[50] Chen Y., Liu J., Zhou B., Yang M., Feng H. (2022). The Value of Ki-67 as a Prognostic Biomarker in Osteosarcoma: A Systematic Review and Meta-Analysis. Asian J. Surg..

[51] Pu Y., Wang J., Wang J., Wang S. (2022). Association of Preoperative Plasma D-Dimer and Fibrinogen and Osteosarcoma Outcome. Front. Oncol..

[52] Sánchez Romero E.A., Lim T., Alonso Pérez J.L., Castaldo M., Martínez Lozano P., Villafañe J.H. (2021). Identifying Clinical and MRI Characteristics Associated with Quality of Life in Patients with Anterior Cruciate Ligament Injury: Prognostic Factors for Long-Term. Int. J. Environ. Res. Public. Health.

[53] Negrini S., Imperio G., Villafañe J.H., Negrini F., Zaina F. (2013). Systematic Reviews of Physical and Rehabilitation Medicine Cochrane Contents. Part 1. Disabilities Due to Spinal Disorders and Pain Syndromes in Adults. Eur. J. Phys. Rehabil. Med..

[54] Garay-Sánchez A., Ferrando-Margelí M., Navarro-Segura M., Fernández-Celaya A., Orejuela-Aparicio E., Cuenca-Zaldivar J.N., Sánchez-Romero E.A., Marcén-Román Y. (2026). Impact and Feasibility of a Group-Based Therapeutic Exercise Program on Strength and Endurance in Hospitalized Patients with Spinal Cord Injury: A Quasi-Experimental Study. J. Neuroeng. Rehabil..

[55] Durández P.C., Sánchez-Romero E.A., Fraga A.N., Benayas C.R., De Viedma R.-H.G., Ferrer D.M., Solana I.G., Nahúm M.C., Sebastián M.V., Guerra R.M.D.L.S. (2025). Toward the Patient Participation Pathway: A Mixed Methods Study of Patients With Cancer and Other Chronic Diseases. Cancer Rep..

