# Supplementary material for: Routine Laboratory Biomarkers for Survival Prediction After Limb-Salvage Surgery in Osteosarcoma: Development of a LASSO-Cox Prognostic Model and Internal Validation
Source: J Clin Med. 2026 Sep 8;15(18):6962. doi: 10.3390/jcm15186962 (PMC13607170; doi:10.3390/jcm15186962)
Supplement: Supplementary file 1 [file jcm-15-06962-s001.zip › jcm-4465976-supplementary.pdf]

Supplementary Figure S1(A-F). Representative imaging and intraoperative images of distal femoral osteosarcoma, demonstrating segmental resection and tumor-type prosthetic replacement.

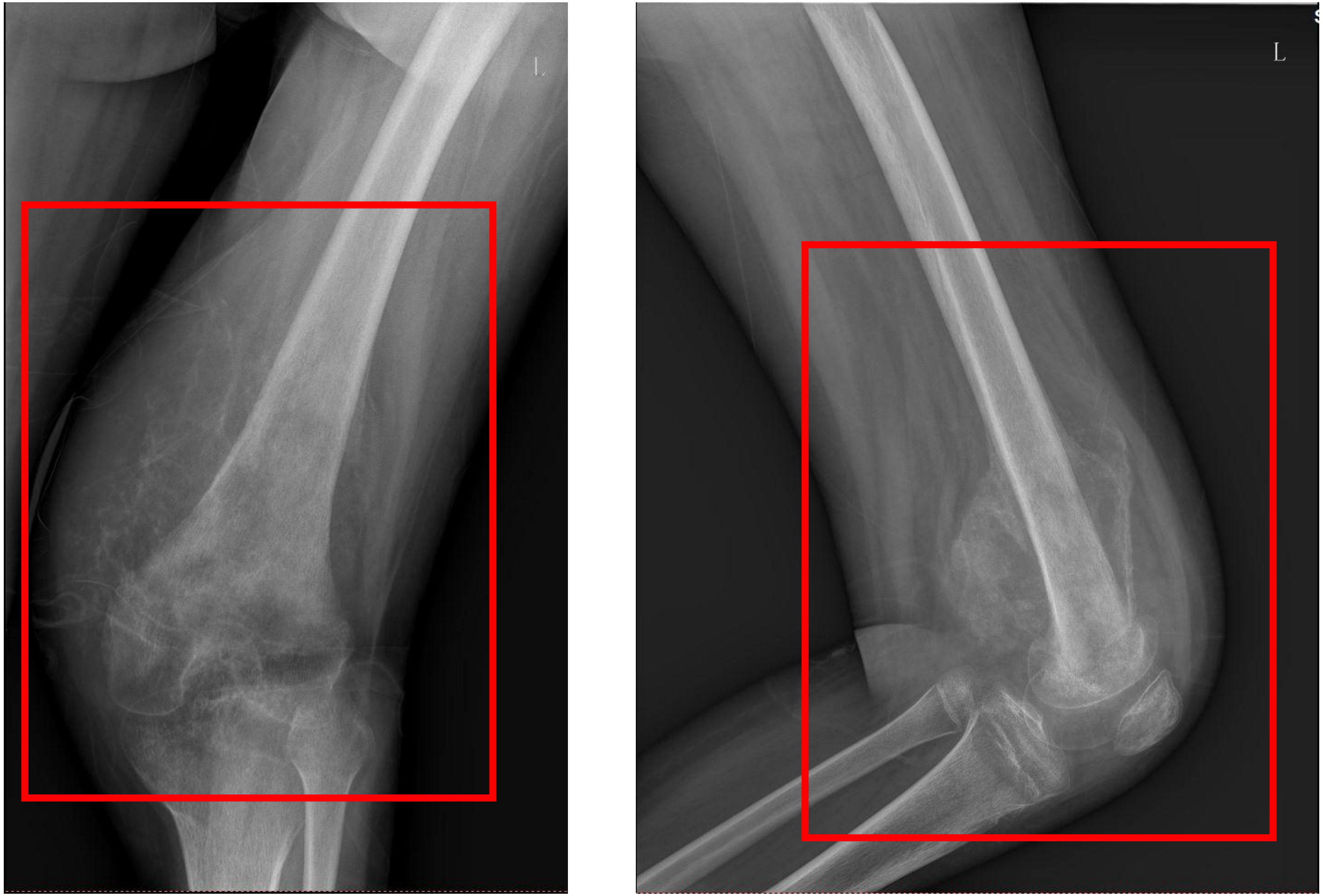

Supplementary Figure S1A1. Preoperative plain radiograph (DR) of the left distal femur showed extensive osteolytic destruction and a large surrounding soft tissue mass.

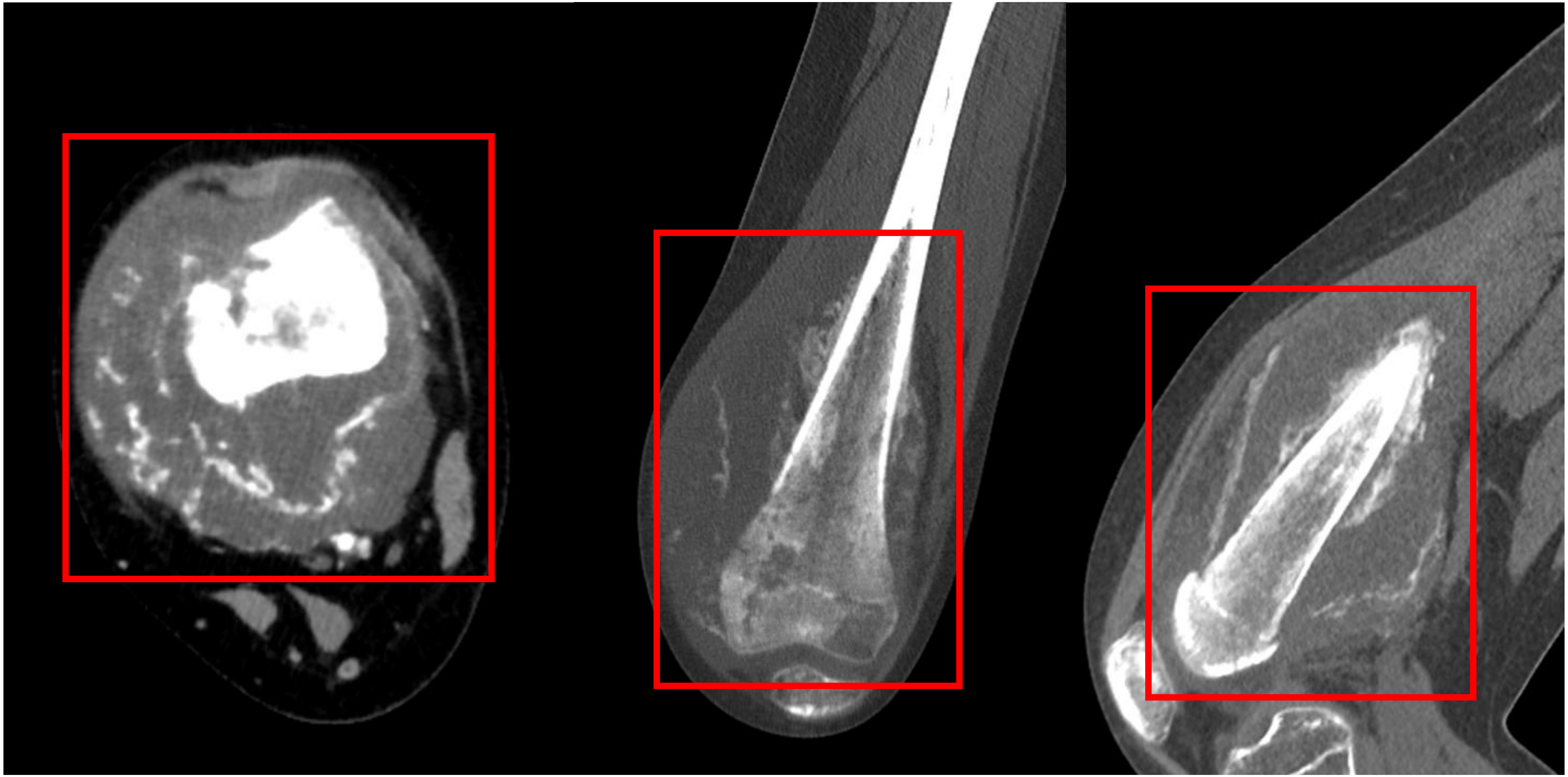

Supplementary Figure S1A2. Preoperative computed tomography (CT) of the left distal femur revealed extensive osteolytic destruction and a large surrounding soft tissue mass.

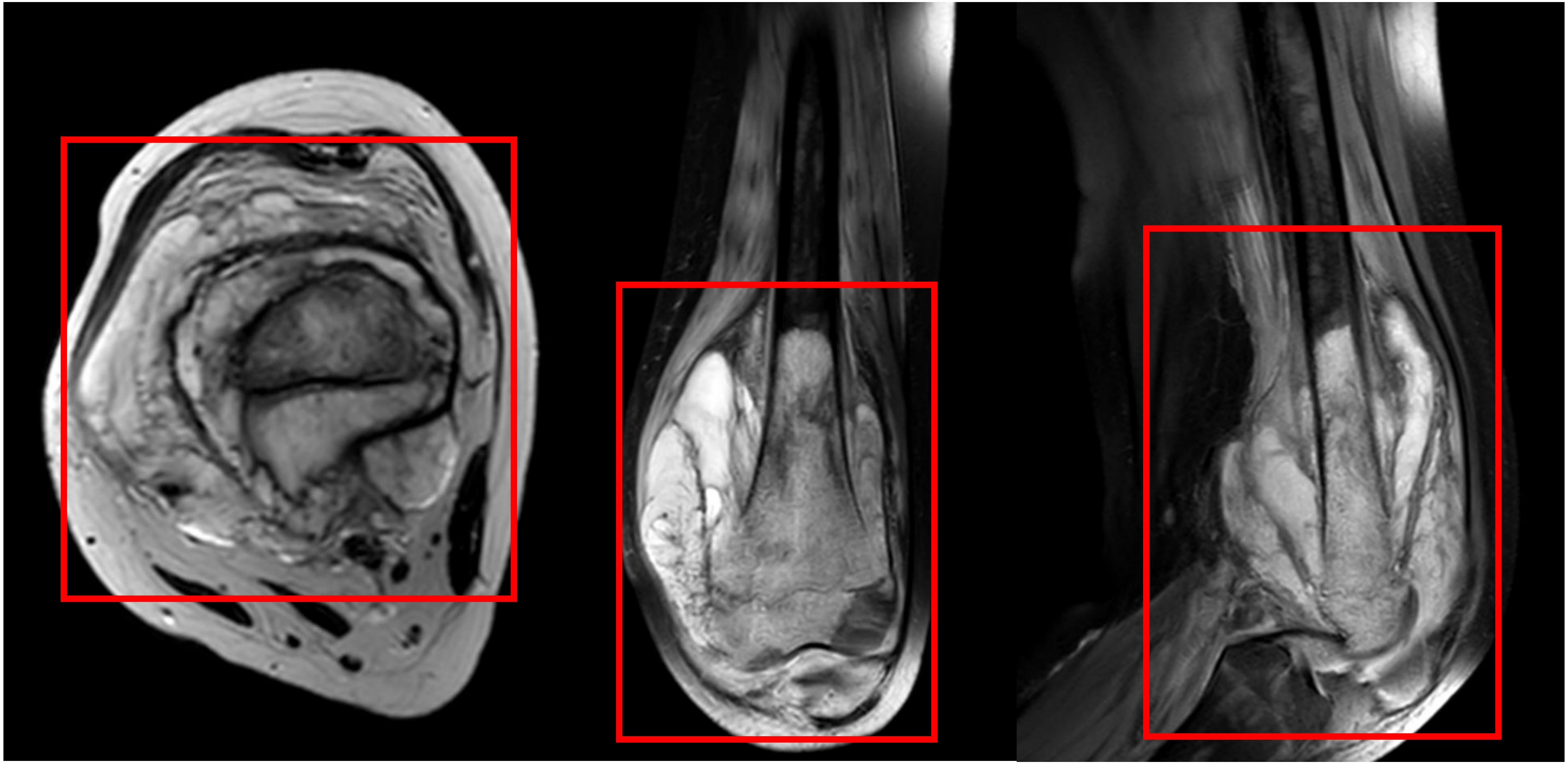

Supplementary Figure S1A3. Preoperative magnetic resonance imaging (MRI) of the left distal femur demonstrated extensive osteolytic destruction and a large surrounding soft tissue mass.

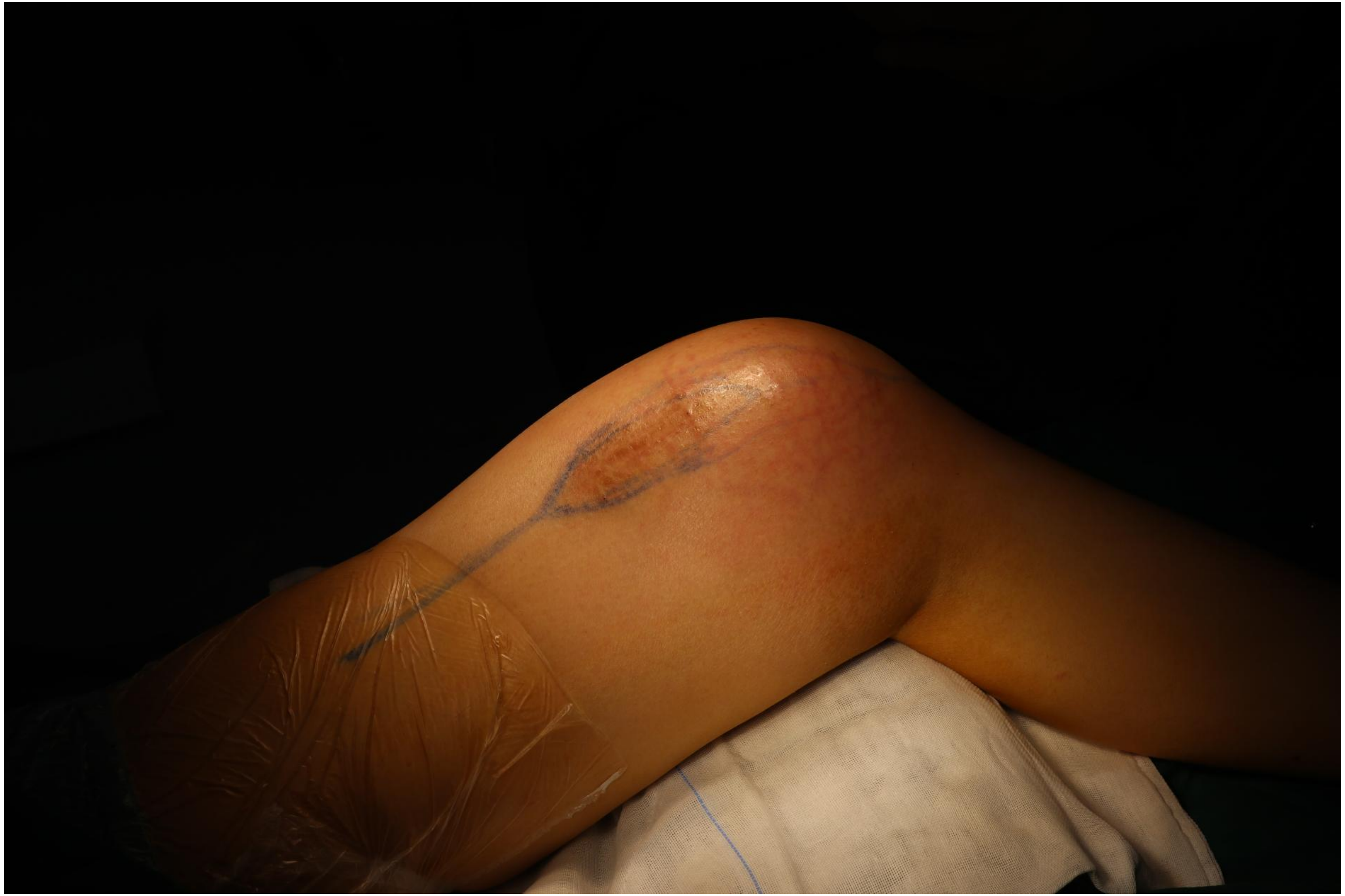

Supplementary Figure S1B1. Surgical exposure: a 32-cm longitudinal spindle-shaped incision was made on the medial aspect of the distal femur.

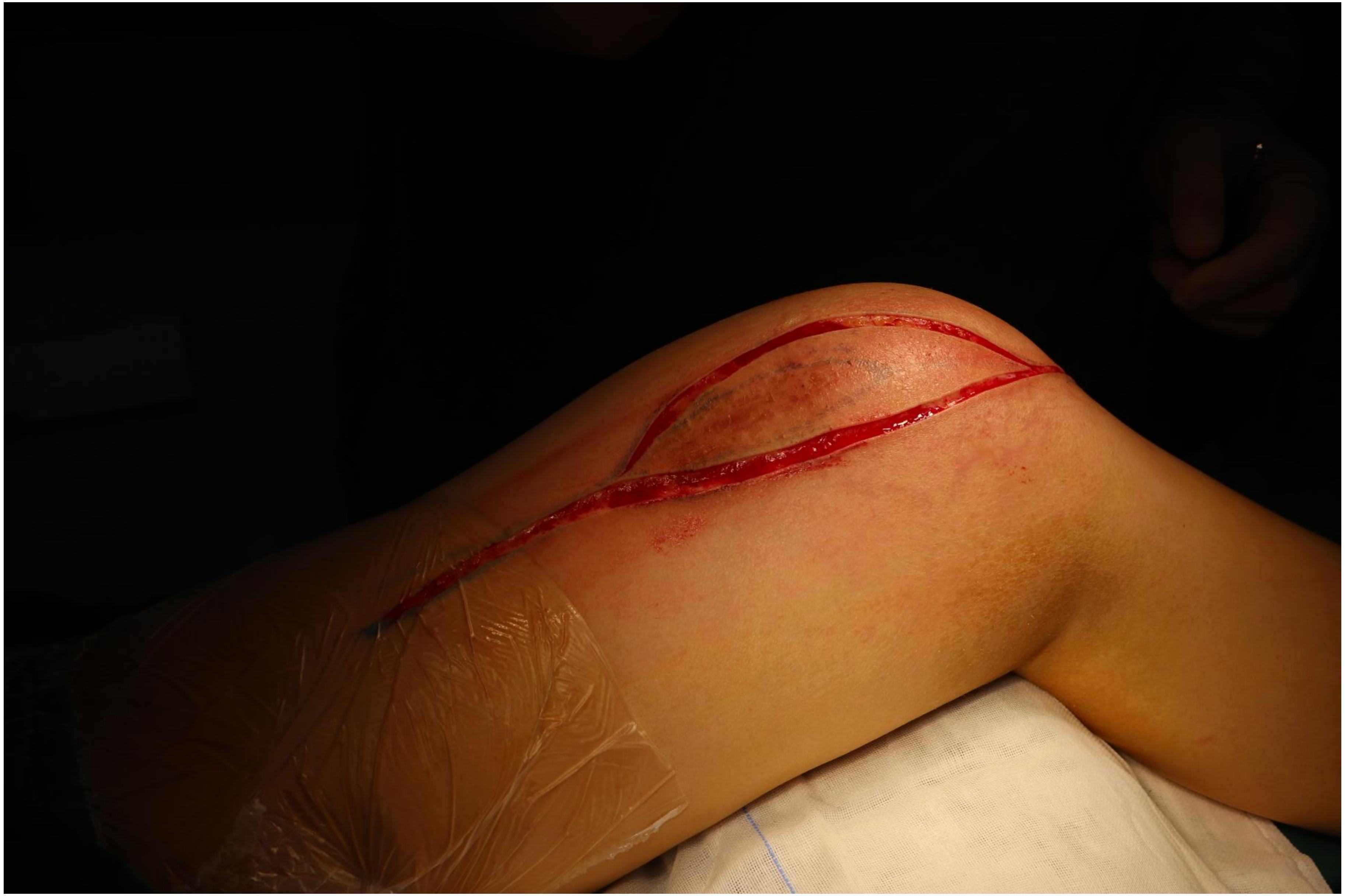

Supplementary Figure S1B2. The previous biopsy tract was excised en bloc.

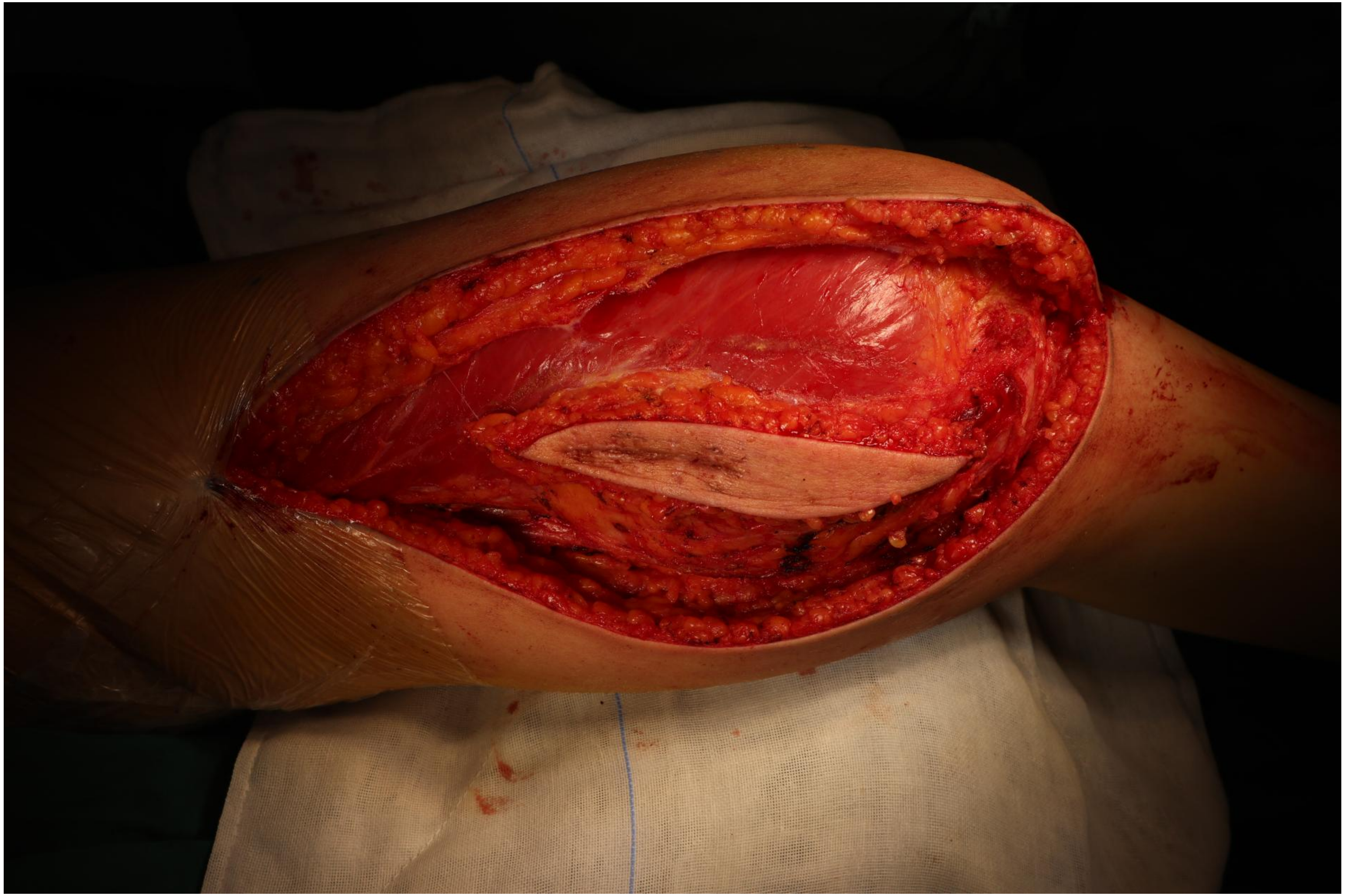

Supplementary Figure S1B3. The skin, subcutaneous tissue, and deep fascia were sequentially incised to expose the vastus medialis muscle.

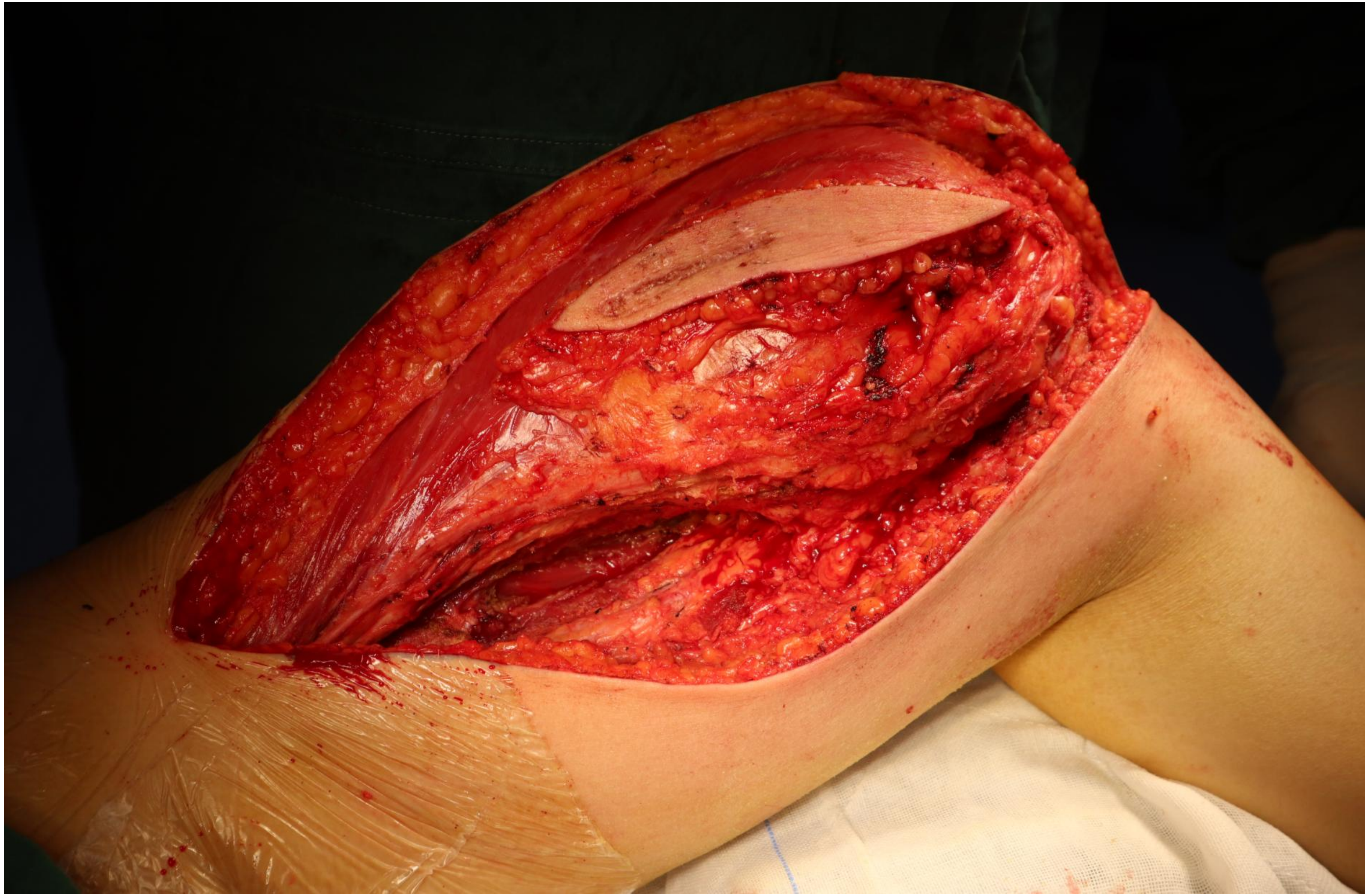

Supplementary Figure S1B4. The distal portion of the vastus medialis was involved, and dissection was carried out through the normal intermuscular plane.

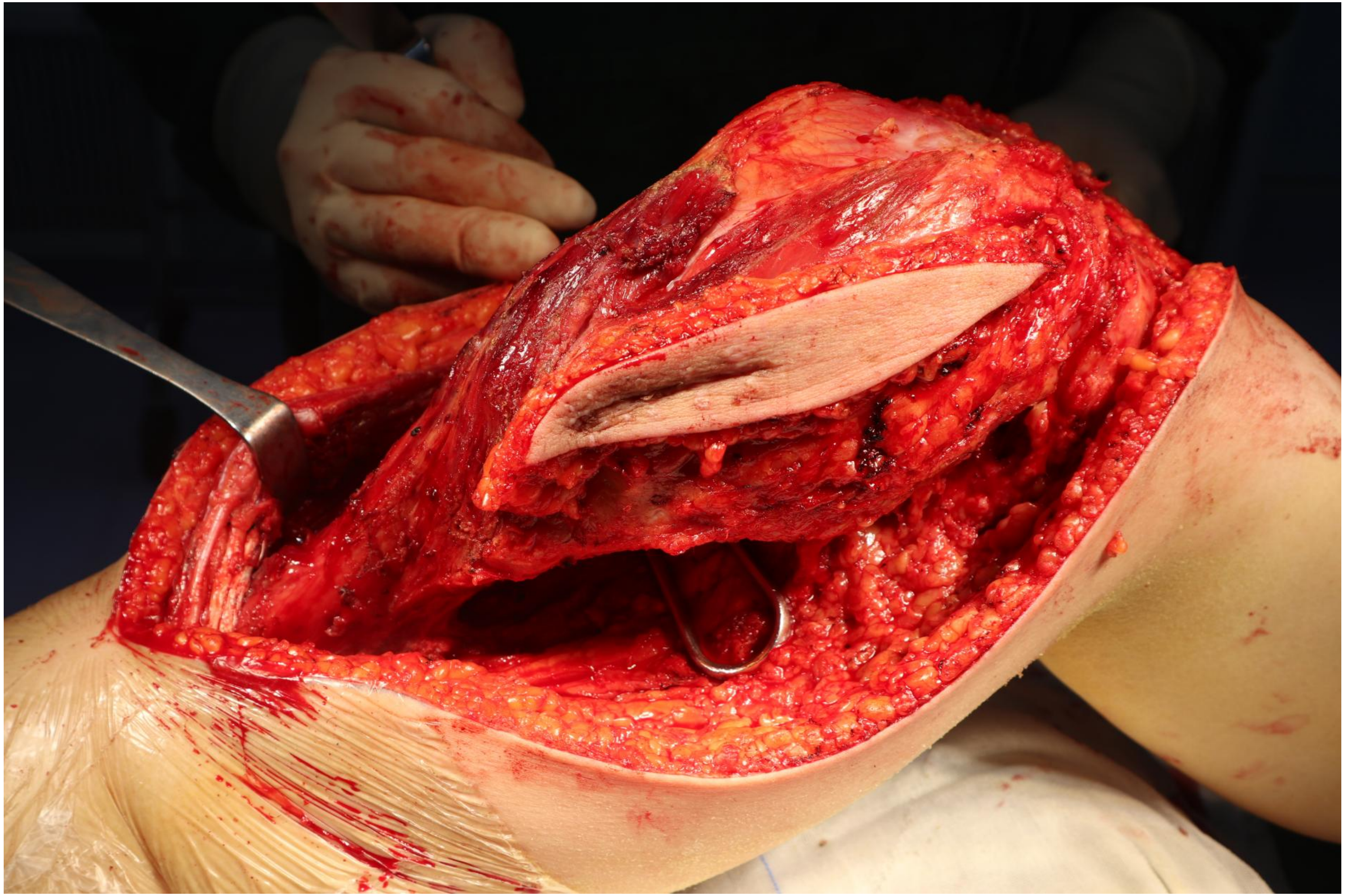

Supplementary Figure S1B5. The dissection was gradually extended to the deeper layer to expose the distal femur.

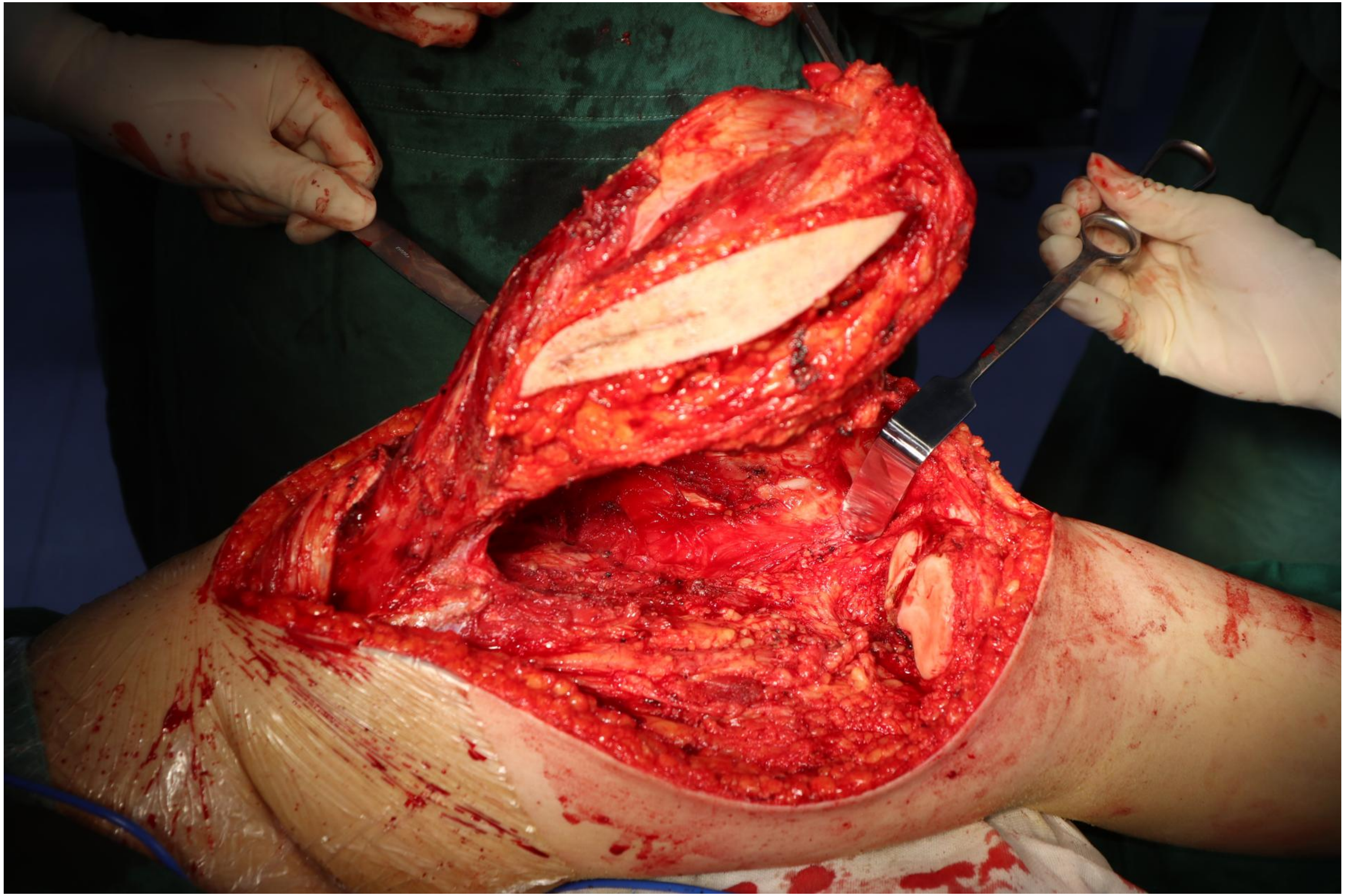

Supplementary Figure S1C1. Tumor resection: the distal femur was fully mobilized, and a segmental osteotomy of approximately 19 cm was designed and performed.

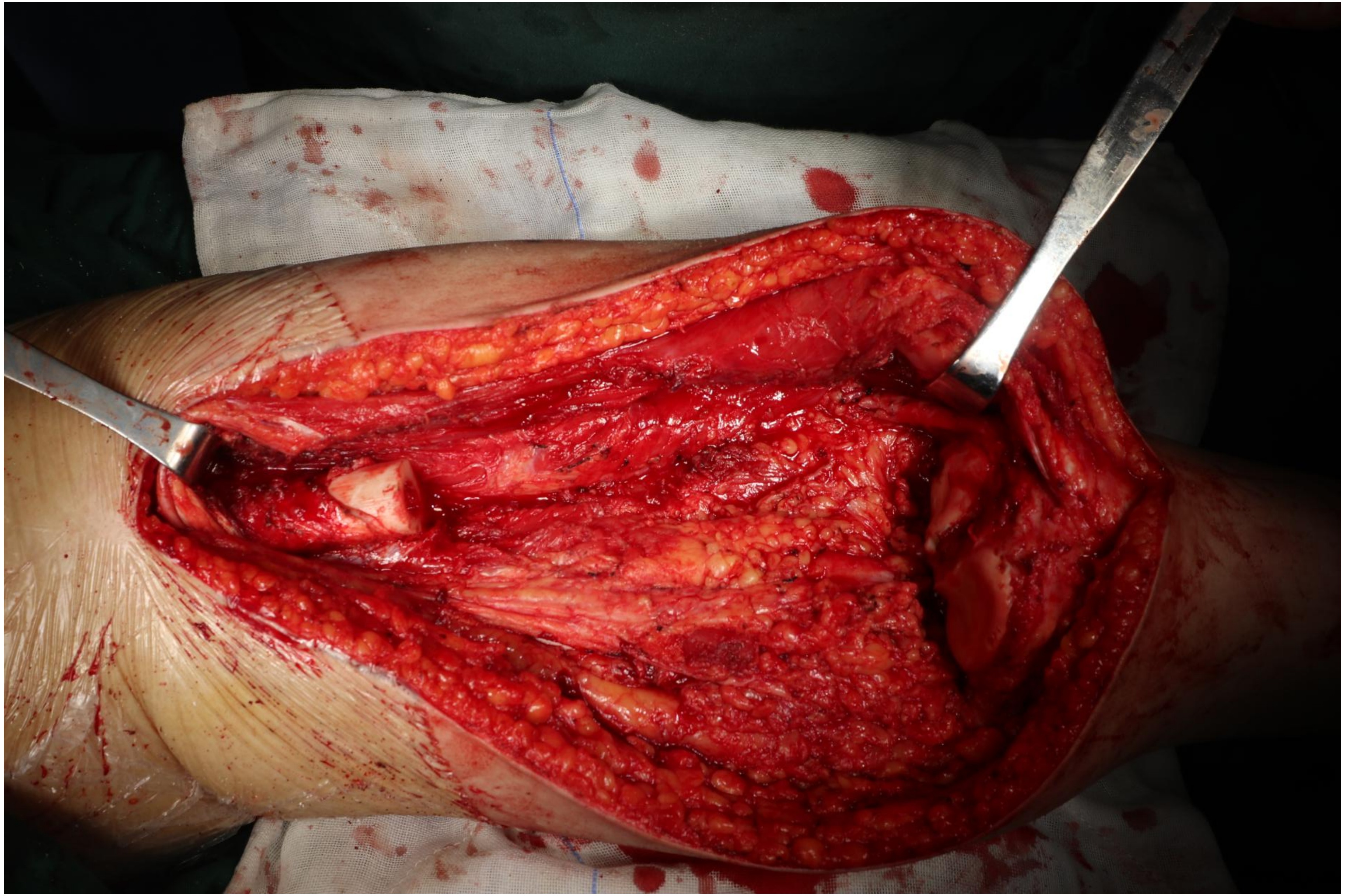

Supplementary Figure S1C2. The tumor was completely resected enbloc with negative margins.

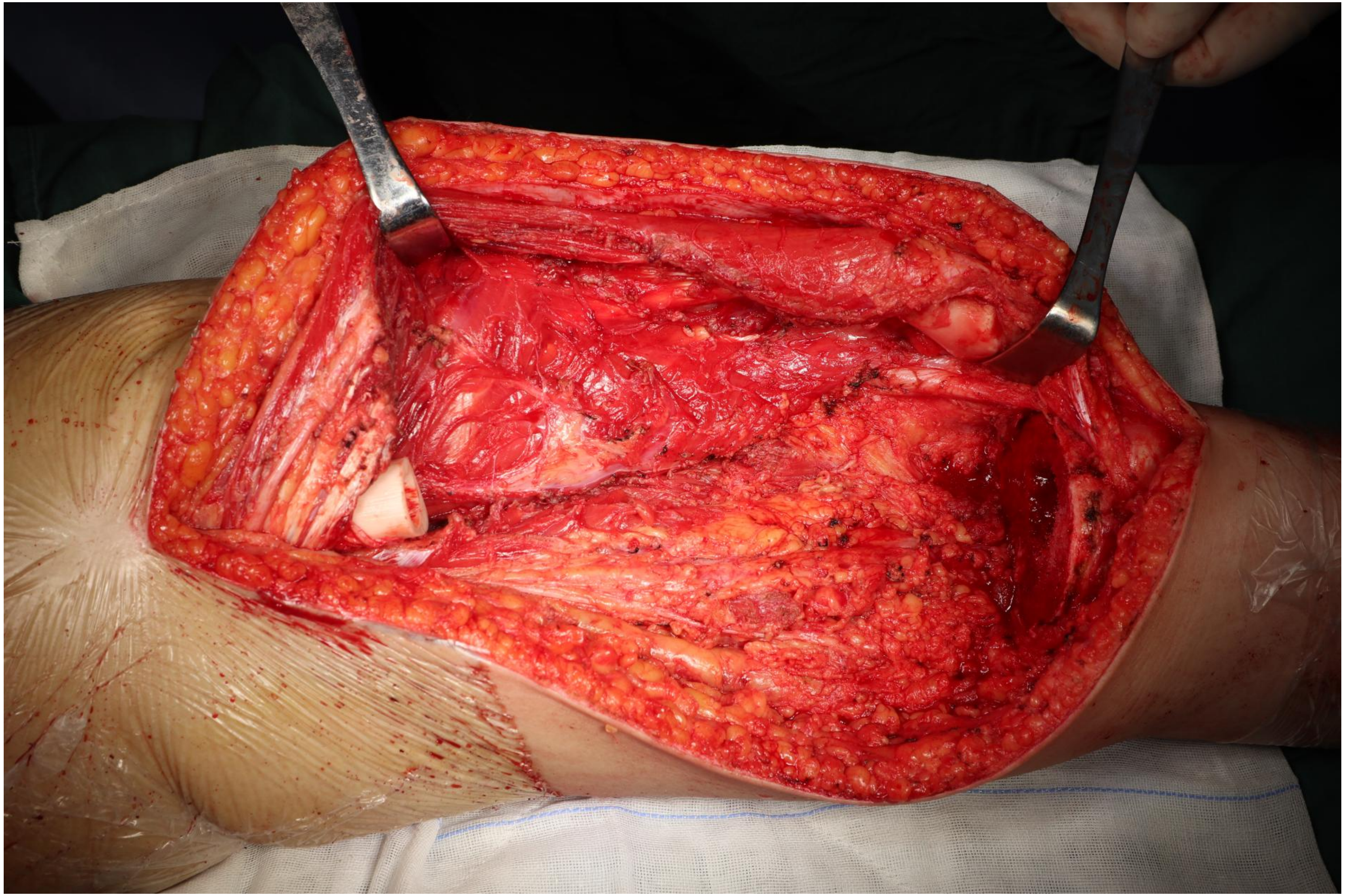

Supplementary Figure S1D1. Prosthetic reconstruction: intramedullary reaming was performed on both the tibial and femoral ends.

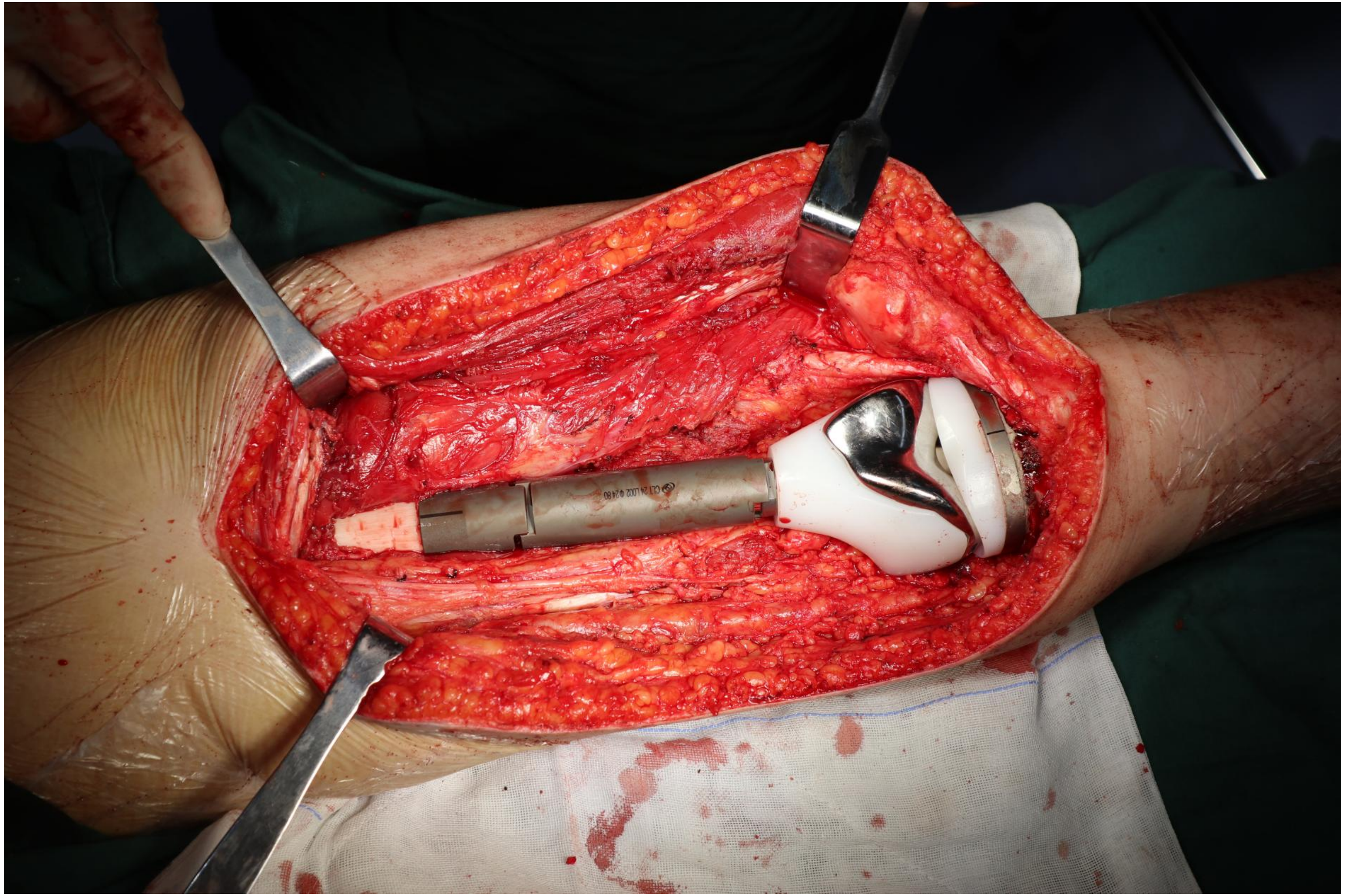

Supplementary Figure S1D2. After trial reduction, the prosthesis was implanted with satisfactory knee joint mobility and equal limb length.

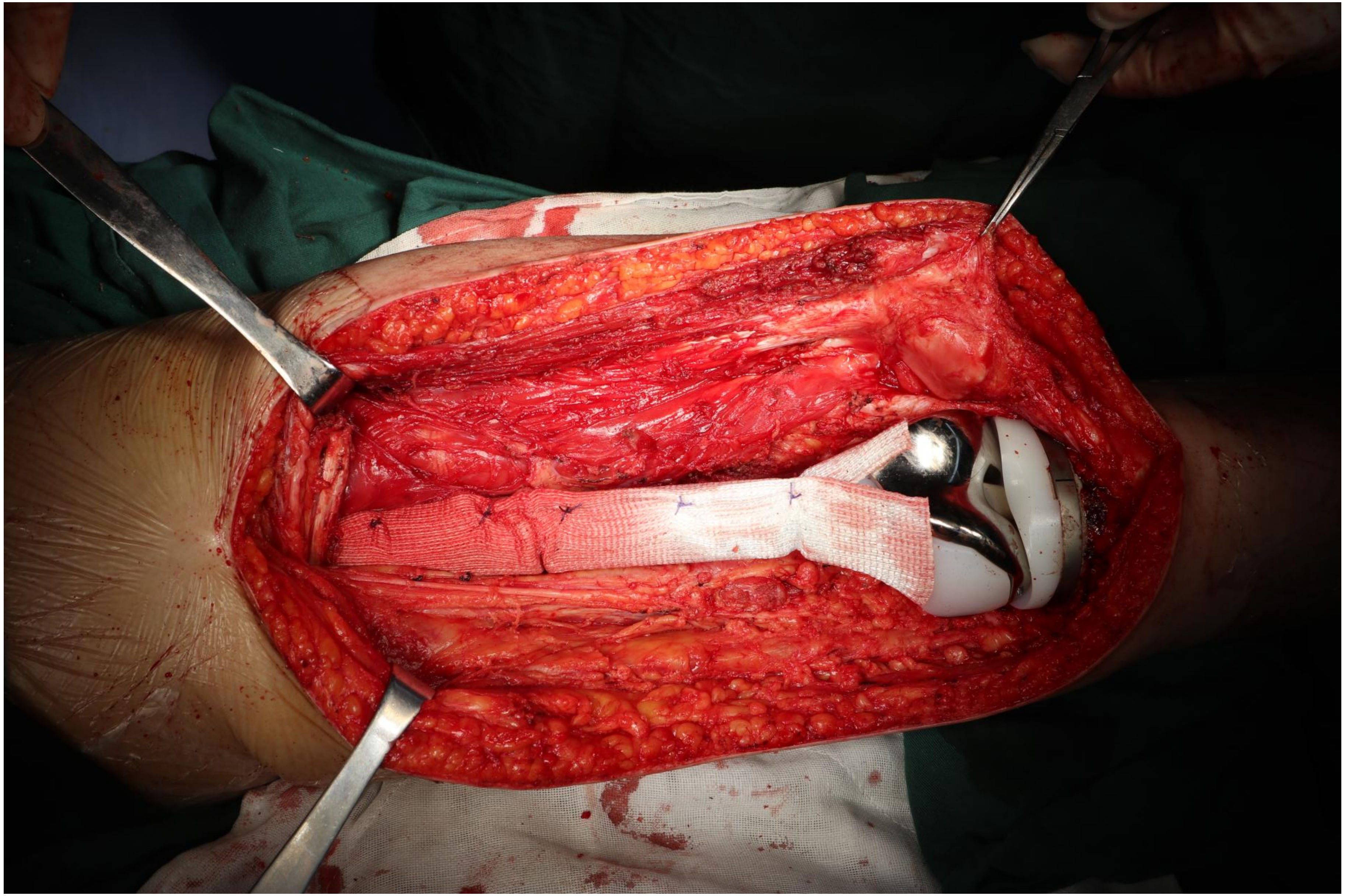

Supplementary Figure S1D3. The prosthetic body was wrapped with a mesh patch and securely sutured.

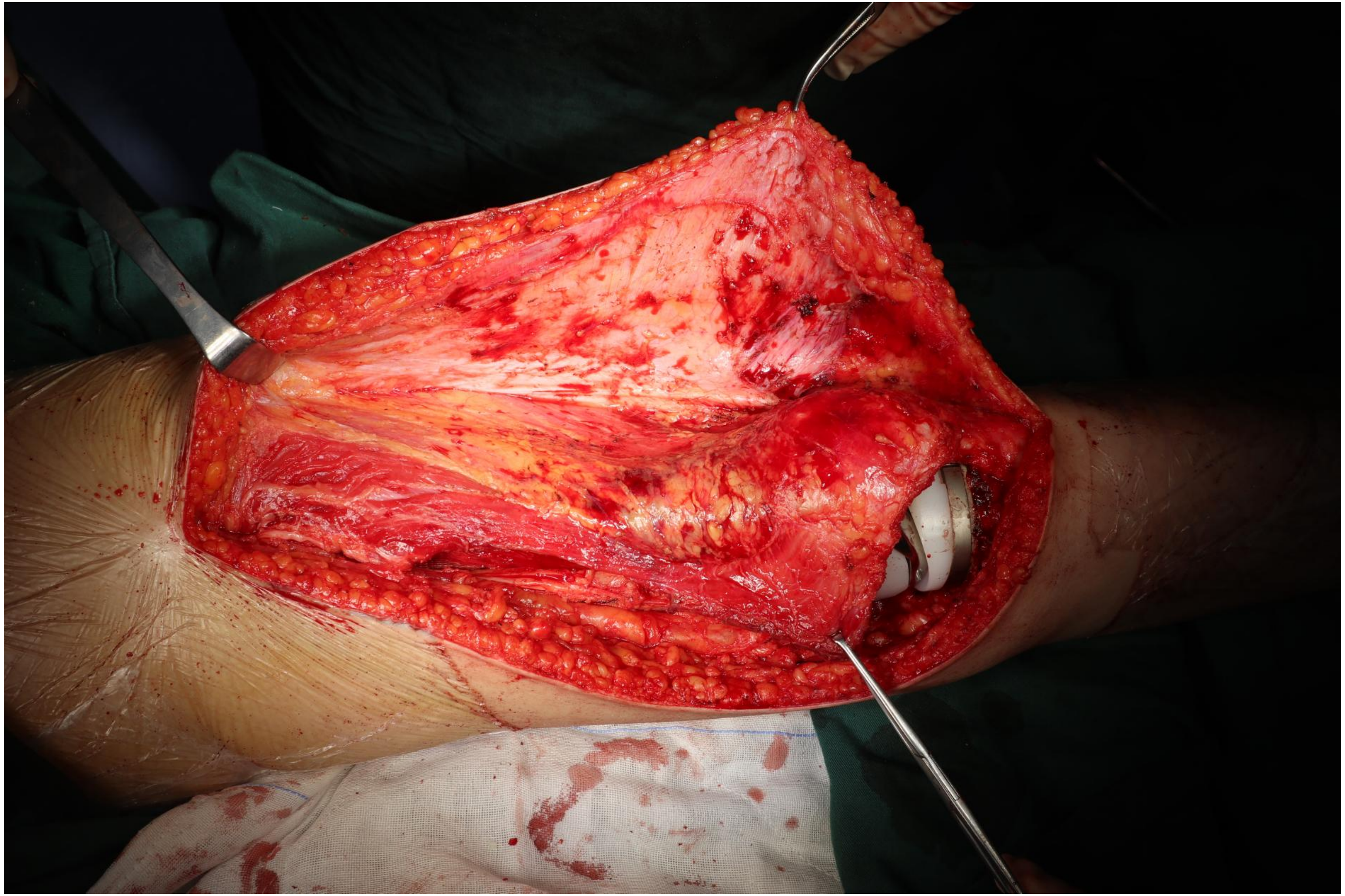

Supplementary Figure S1D4. The muscles were reattached and sutured onto the ligaments over the prosthetic surface.

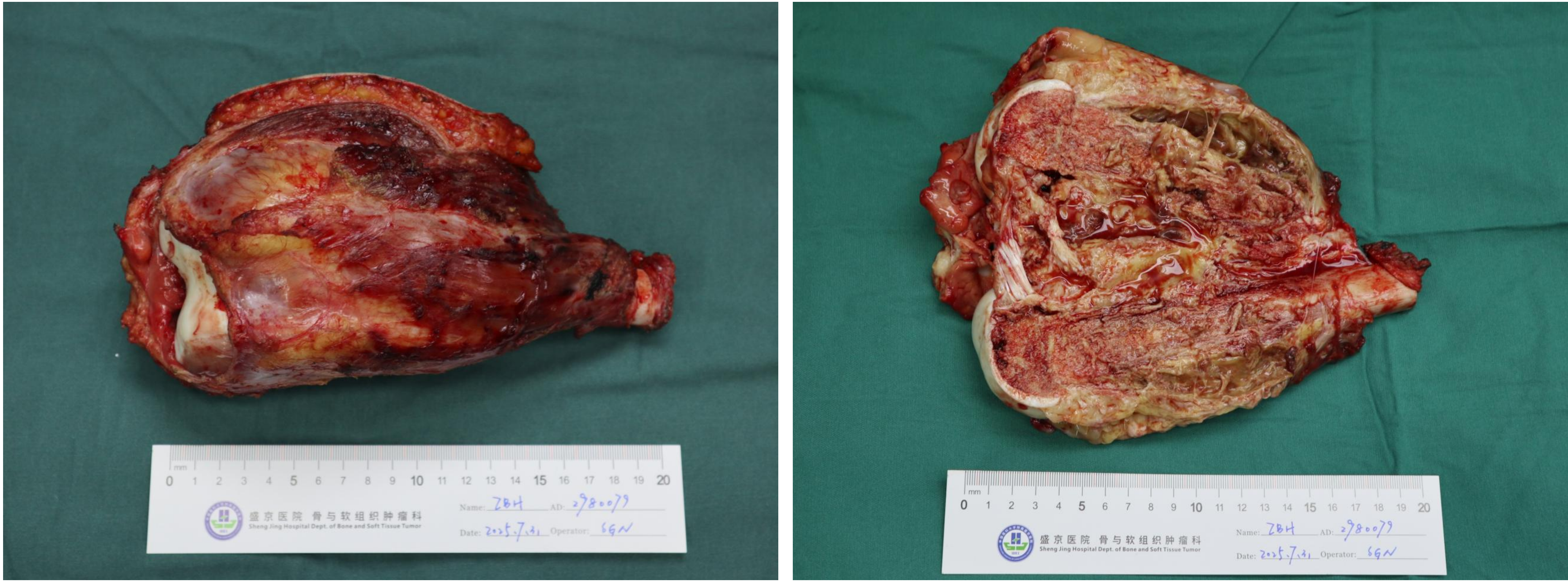

Supplementary Figure S1E. Gross appearance of the resected specimen.

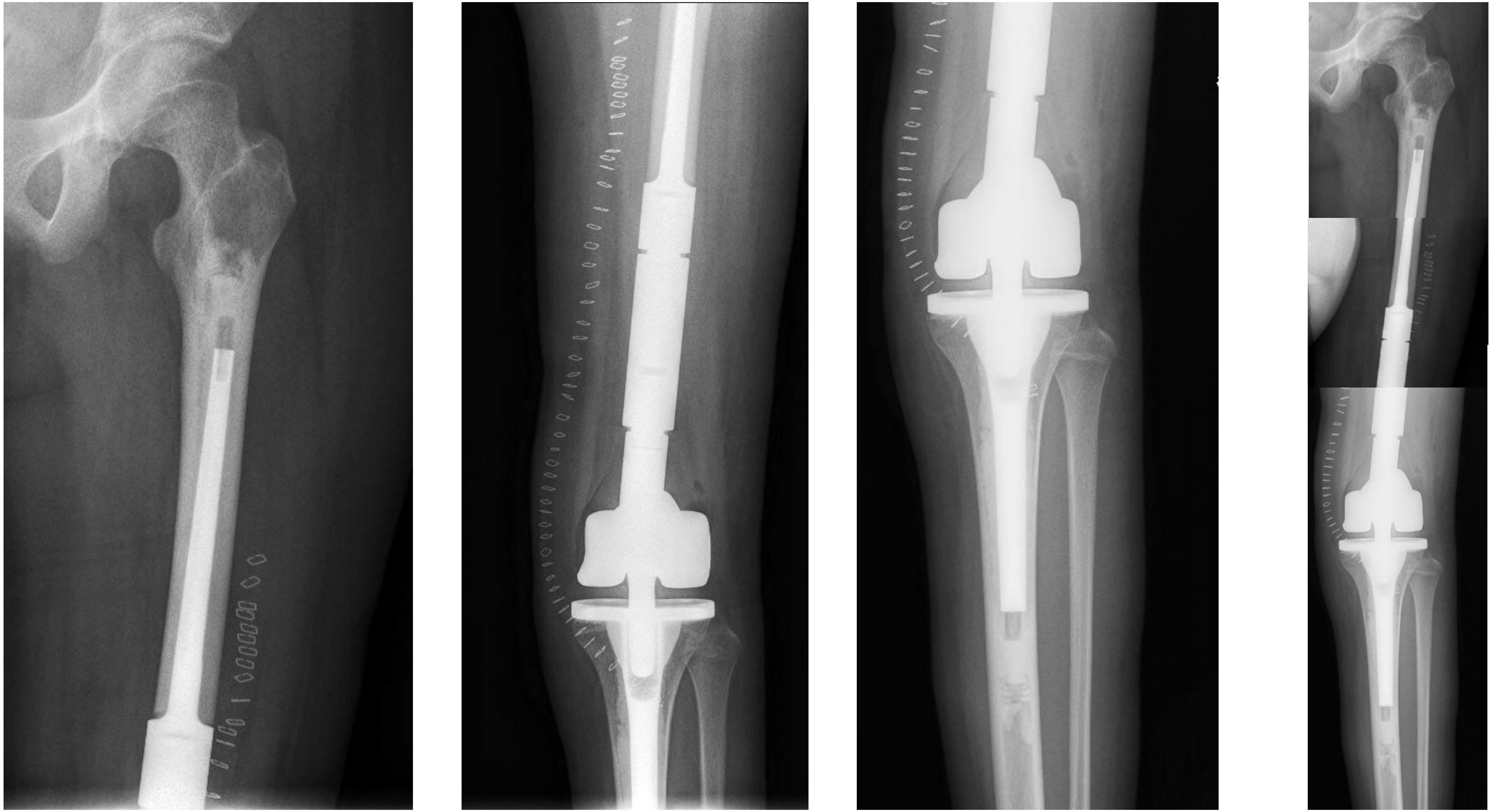

Supplementary Figure S1F. Postoperative plain radiograph (DR) confirmed the satisfactory position of the prosthesis.

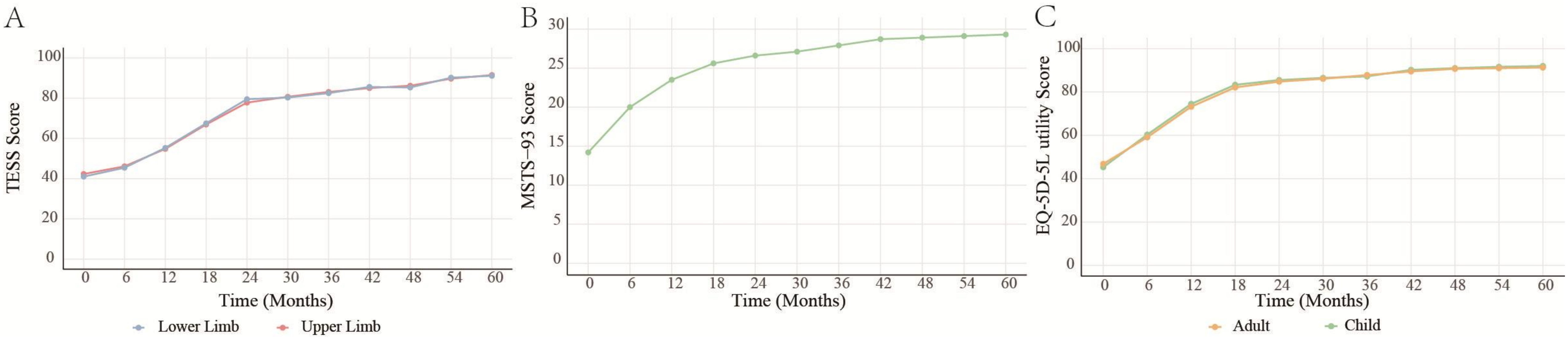

Supplementary Figure S2. Exploratory longitudinal assessment of postoperative functional status and health-related quality of life. A TESS scores over time, with the upper-limb and lower-limb versions plotted together for direct comparison. B EQ-5D-5L utility scores over time, with health states converted into utility scores using the Chinese mainland EQ-5D-5L value set developed by Luo et al. C MSTS-93 scores over time.

Data points represent mean scores at each postoperative time point (months). Different lines distinguish the respective versions within panel A or indicate the corresponding outcome measure in panels B and C. Shaded areas and error bars are not shown because only mean values were available.

Variable	Schoenfeld χ^2	P value
ALP	1.374	0.241
LDH	3.496	0.062
FIB	3.185	0.074
D-dimer	3.271	0.071
**Global test**	**9.825**	**0.044**

Supplementary Table S1. Proportional-hazards assumption test for the final four-biomarker Cox model. The proportional-hazards assumption was assessed using Schoenfeld residuals. P values >0.05 indicate no statistically significant evidence of violation for individual covariates. The global test was marginally significant (P = 0.044), suggesting a potential departure from the proportional-hazards assumption at the overall model level.

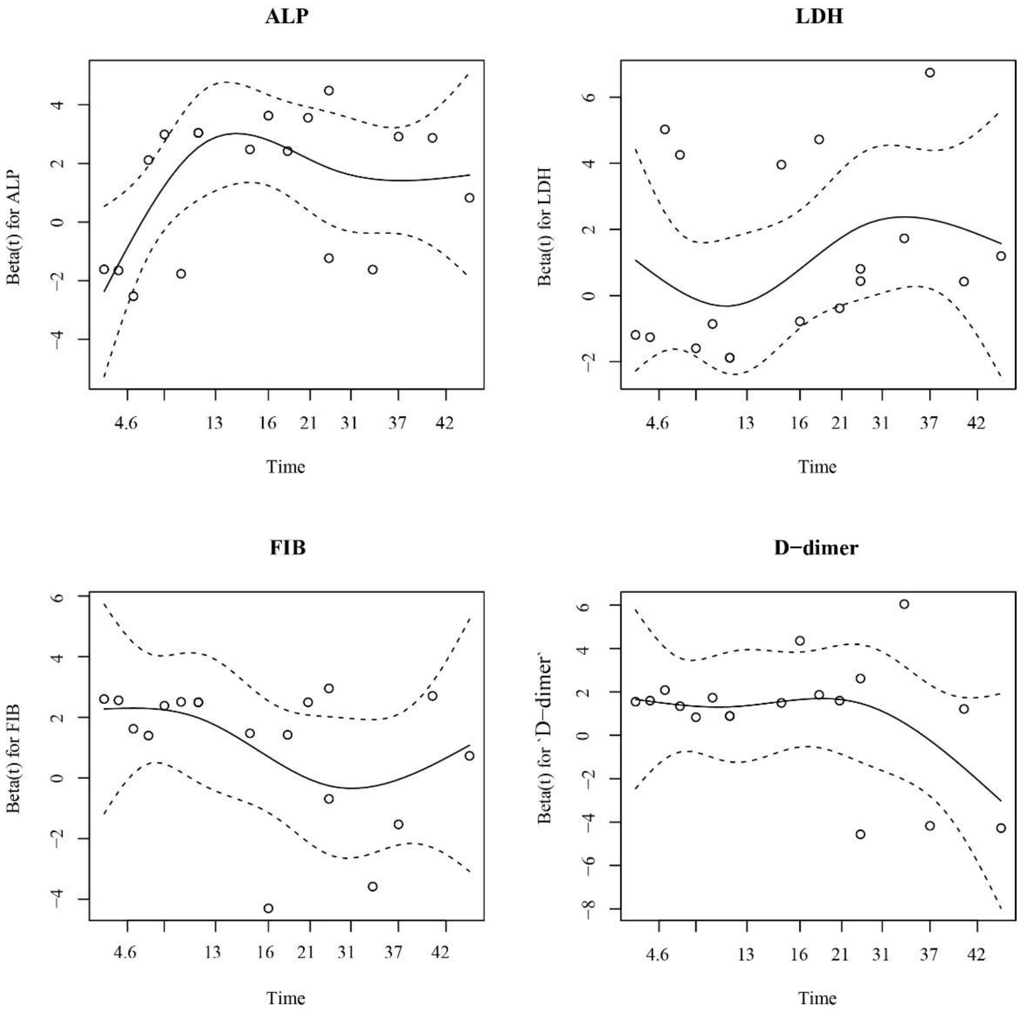

Supplementary Figure S3. Schoenfeld residual-based assessment of the proportional-hazards assumption for individual biomarkers. Schoenfeld residual plots are shown for ALP, LDH, fibrinogen (FIB), and D-dimer. The solid lines represent the estimated time-varying effects, with shaded areas indicating the corresponding 95% confidence intervals. No individual biomarker showed statistically significant evidence of a proportional-hazards violation (ALP, P = 0.241; LDH, P = 0.062; FIB, P = 0.074; D-dimer, P = 0.071). Although the global Schoenfeld test was marginally significant (P = 0.044), this finding was interpreted as indicating possible model-level departure from proportionality rather than a definitive violation by any individual biomarker.

Predictor	Candidate	Selection frequency	Selected in final model	LASSO coefficient	PH P value
ALP	Yes	0.94	Yes	1.290766	0.241
LDH	Yes	0.80	Yes	0.731692	0.062
FIB	Yes	0.99	Yes	0.827207	0.074
D-dimer	Yes	0.92	Yes	1.033803	0.071
Ki-67	Yes	0.40	No	0	—
Age	Yes	0.41	No	0	—
Tumor size	Yes	0.40	No	0	—
Lung metastasis	Yes	0.64	No	0	—
Sex	Yes	0.56	No	0	—

Supplementary Table S2. Predictor selection, LASSO coefficients, and proportional-hazards assessment. Selection frequency was estimated from 1000 bootstrap resamples using 10-fold cross-validation with the lambda.min criterion. Predictors with a selection frequency ≥0.80 were considered stable and retained in the final penalized LASSO-Cox model. LASSO coefficients represent the penalized regression coefficients from the final model; a coefficient of 0 indicates that the predictor was not retained. Proportional-hazards assumptions were assessed using Schoenfeld residuals, with the reported P values corresponding to the individual predictor tests. **P < 0.05 was considered statistically significant.** Selection frequency and LASSO coefficients were used for model development and stability assessment and should not be interpreted as evidence of independent clinical prognostic significance.

Item	Standard formula / definition
LASSO-Cox model	$h(t \mid X) = h0(t) * \exp(LP)$
LASSO objective	Minimize { -log-likelihood + lambda * sum(abs(coefficient)) }
Linear predictor (LP)	LP = X1*B1 + X2*B2 + ... + Xp*Bp
Relative risk	RR = exp(LP)
Predicted survival	$S(t \mid X) = S0(t) \wedge \exp(LP)$
Predicted mortality risk	$Risk(t) = 1 - S(t \mid X)$
Selection frequency	Selection frequency = number of bootstrap samples with non-zero coefficient / total bootstrap samples
Stability criterion	Selection frequency >= 0.80
Lambda selection	10-fold cross-validation; lambda.min

Supplementary Table S3. Final LASSO-Cox model specification. The final prognostic model was developed using LASSO-Cox regression with 10-fold cross-validation and the lambda.min criterion. The final model included ALP, D-dimer, LDH, and FIB. LASSO coefficients were obtained directly from the penalized model without subsequent unpenalized Cox refitting. Predictors with a bootstrap selection frequency of at least 0.80 were considered stable.

Measure	Standard formula / definition
Harrell's C-index	C = proportion of concordant comparable pairs
Time-dependent AUC	AUC(t) = probability that a higher predicted risk is assigned to a patient who experiences the event by time t than to a patient who remains event-free beyond t
IPCW Brier score	Brier(t) = mean of the IPCW-weighted squared difference between observed and predicted event status at time t
Bootstrap optimism	Optimism = performance in bootstrap sample - performance when the bootstrap model is applied to the original cohort
Mean optimism	Mean optimism = sum of bootstrap optimism / number of valid bootstrap samples
Optimism-corrected performance	Corrected performance = apparent performance - mean optimism

Supplementary Table S4. Definitions of discrimination and prediction error measures. Harrell's C-index was used to assess overall concordance across the follow-up period. Time-dependent AUCs were defined at prespecified time horizons while accounting for right censoring. IPCW Brier scores were used to quantify prediction error, with lower values indicating better prediction accuracy. Bootstrap optimism was calculated as the difference between performance in the bootstrap sample and performance when the bootstrap model was applied to the original cohort. Optimism-corrected performance was calculated by subtracting mean optimism from apparent performance.

Measure	Standard formula / definition
Calibration slope	Observed outcome = intercept + calibration slope * predicted linear predictor
Calibration-in-the-large	Calibration intercept represents systematic over- or underprediction of risk
Sensitivity	Sensitivity = TP / (TP + FN)
Specificity	Specificity = TN / (TN + FP)
PPV	PPV = TP / (TP + FP)
NPV	NPV = TN / (TN + FN)
Individual PH test	Schoenfeld residual test for whether the predictor effect remains constant over time
Global PH test	Schoenfeld residual test for the proportional-hazards assumption across all predictors

Supplementary Table S5. Definitions of calibration, classification, and proportional-hazards assessment. Calibration measures were used to assess agreement between predicted and observed risk. Sensitivity, specificity, positive predictive value (PPV), and negative predictive value (NPV) were evaluated at the prespecified 60-month horizon with adjustment for right censoring. The proportional-hazards assumption was assessed using Schoenfeld residual tests for individual predictors and globally. These measures were considered internal and exploratory because of the limited sample size and number of events.
